# Diversity of *Neofusicoccum parvum* for the Production of the Phytotoxic Metabolites (-)-Terremutin and (*R*)-Mellein

**DOI:** 10.3390/jof8030319

**Published:** 2022-03-19

**Authors:** Patricia Trotel-Aziz, Guillaume Robert-Siegwald, Olivier Fernandez, Catarina Leal, Sandra Villaume, Jean-François Guise, Eliane Abou-Mansour, Marc-Henri Lebrun, Florence Fontaine

**Affiliations:** 1 Research Unit Résistance Induite et Bioprotection des Plantes, Université de Reims Champagne-Ardenne, RIBP EA 4707, INRAE USC 1488, SFR Condorcet FR CNRS 3417, 51100 Reims, France; olivier.fernandez@univ-reims.fr (O.F.); catarina.da-cunha-maia-leal@univ-reims.fr (C.L.); sandra.villaume@univ-reims.fr (S.V.); jean-francois.guise@univ-reims.fr (J.-F.G.); florence.fontaine@univ-reims.fr (F.F.); 2 Independent Researcher, 49000 Angers, France; guillaume.robert.siegwald@gmail.com; 3 Département de biologie végétale, Université de Fribourg, Chemin du Musée 10, 1700 Fribourg, Switzerland; eliane.abou-mansour@unifr.ch; 4 Research Group Genomics of Plant-Pathogen Interactions, Research Unit Biologie et Gestion des Risques en Agriculture, UR 1290 BIOGER, Université Paris Saclay, INRAE, Avenue Lucien Brétignières, 78850 Thiverval-Grignon, France; marc-henri.lebrun@inrae.fr

**Keywords:** *Vitis vinifera* cv. chardonnay, fungal plant pathogen, virulence factors, plant phenotyping, plant defense responses, pathogen genotyping, Np-Bt67, Np-B, mutant, secondary metabolism

## Abstract

Two *Neofusicoccum* *parvum* isolates and a UV mutant were characterized for their phytotoxin production in vitro, their pathogenicity on grapevine, and their genome sequenced. The isolate Np-Bt67 produced high level of (-)-terremutin, but almost no (*R*)-mellein, and it was the most aggressive on grapevine, triggering apoplexy. Similar symptoms were not induced by purified (-)-terremutin. The isolate Bourgogne S-116 (Np-B) produced 3-fold less (-)-terremutin and high amounts of (*R*)-mellein, but it was less aggressive on grapevine than Np-Bt67. The UV9 mutant obtained from Np-B (NpB-UV9) no longer produced (-)-terremutin but overproduced (*R*)-mellein by 2.5-fold, and it was as pathogenic as its parent. NpB-UV9 differed from its parent by simple mutations in two genes (transcription factor *UCR-NP2_6692*, regulatory protein *UCR-NP2_9007*), not located neither near (*R*)-mellein, nor (-)-terremutin biosynthetic genes, but likely involved in the control of (-)-terremutin biosynthesis. Grapevine immunity was disturbed upon challenge with these pathogens or purified phytotoxins, leading to an upregulation of SA-dependent defenses, while (-)-terremutin interfered with host JA/ET-dependent defenses. Our results suggest that neither (-)-terremutin nor (*R*)-mellein alone is essential for the pathogenicity of *N. parvum* on grapevine, since isolate/mutant non-producing these toxins in vitro is pathogenic. However, these phytotoxins could play a quantitative role in the infection process.

## 1. Introduction 

*Botryosphaeria* dieback caused by fungi from the *Botryosphaeriaceae* family is a serious disease in young [1] and established vineyards worldwide [2,3,4]. The main disease symptoms are stem canker and necrosis. Severe infection, namely apoplexy, is due to increased internal longitudinal necrotic lesions leading to the occurrence of typical foliar symptoms with discolorations and finally to a full dead branch [5,6,7,8,9,10,11]. The severity of disease symptoms varied with grapevine cultivars, inoculated tissues, and abiotic conditions [9,12,13,14,15,16]. These symptoms are thought to result from the synergistic actions of the plant cell wall-degrading enzymes and phytotoxic metabolites produced by these fungi [17,18,19,20]. In particular, because these fungi are only detected in the wood, but never in symptomatic leaves [21], phytotoxins are suspected to be involved in the production of foliar symptoms once transported from the infection site to the leaves via the xylem [22]. Several phytotoxic secondary metabolites produced by *Botryosphaeriaceae* species have been identified, but their precise roles in the infection process are still unclear [17,18,20,21,23,24,25,26,27,28]. 

*Neofusicoccum parvum* is a highly aggressive plant pathogenic fungus of the *Botryosphaeriaceae* family [1,9]. *N. parvum* isolates from grapevine produce in vitro (-)-terremutin and its derivatives [17,20,23], as well as (*R*)-mellein [17,20,21,23], although the production levels of these two major toxins vary according to isolates [23]. To date, (*R*)-mellein and the (-)-terremutin precursor, 6-methyl salicylic acid (6-MSA), were both identified in infected wood and green shoots of grapevine expressing *Botryosphaeria* dieback or apoplectic symptoms [17,20,23,29,30]. The corresponding biosynthetic pathways of these phytotoxins are known in other fungi [31,32,33,34]. In *Aspergillus terreus*, (-)-terremutin is biosynthesized from 6-MSA by different enzymes [34]. The first step of the pathway involves the *AtX*-encoded PKS producing 6-MSA from acetyl-CoA and malonyl-CoA. 6-MSA is modified by the *AtA*-encoded 6-MSA 1-monooxygenase catalyzing the decarboxylative hydroxylation of 6-MSA to produce 3-methylcatechol. This precursor is modified by the *AtE*-encoded cytochrome P450-monooxygenase catalyzing its hydroxylation leading to 3-methyl-1,2,4-benzenetriol. A cytochrome P450-monooxygenase encoded by *AtG* is suspected to assist AtE in this hydroxylation. 3-methyl-1,2,4-benzenetriol is then modified by the *AtD*-encoded cyclooxygenase catalyzing its epoxidation and hydroxyl oxidation leading to terremutin. In *A. terreus*, terremutin is finally transformed by the *AtC*-encoded GMC oxydoreductase into terreic acid. The genes encoding these enzymes are localized in a cluster and their coordinated transcription is controlled by the transcription factor (TF) *AtF* [32,33,34] localized in this cluster. The (-)-terremutin cluster also contains *atB*, encoding for a putative major facilitator superfamily (MFS) transporter [33]. In *Parastagonospora nodorum*, (*R*)-Mellein biosynthetic pathway requires the PKS (*R*)-mellein synthase encoded by *SNOG_00477* [31]. The PKS encoding genes corresponding to *AtX* and *SNOG_00477* have been identified in the genome of *N. parvum* [31,35,36]. These two phytotoxins are described to induce necrosis on host plant leaf discs of *Vitis vinifera* cv. Chardonnay, Pinot noir, Chasselas, and non-host plant leaves of *Arabidopsis thaliana* [17,20,23,25]. (*R*)-mellein also induces necrosis on grapevine calli and inhibits the growth of wheat embryo culture [11,12,20]. However, the total extracellular compounds produced by *N. parvum* induce more necrosis on Chardonnay calli than (*R*)-mellein alone, suggesting that other fungal metabolites are involved in inducing necrosis [11,12,23]. The (*R*)-mellein derivative methylmellein has a strong antigerminative effect on garden cress [31]. 6-hydroxymellein and its derivative (+)-terrein have phytotoxic effects leading to necrotic lesions on plant leaves and fruits [26,30,37,38]. (*R*)-mellein and its derivative display antimicrobial activities [30]. The (-)-terremutin precursor 6-methylsalicylic acid (6-MSA) and its derivatives, such as terreic acid and m-cresol, have antibacterial and antifungal properties, as well as necrotizing activity on plant leaf tissues [11,17,20,23,39,40,41,42,43]. In mammals, terreic acid affects cellular immunity [33,44]. Finally, both (*R*)-mellein and (-)-terremutin inhibits grapevine defenses induced by biocontrol agents [18]. 

To date, despite the detection of large amounts of (*R*)-mellein in infected grapevine woods correlated with the amount of *N. parvum* detected by qPCR [30], there is no report evaluating the role of (*R*)-mellein and (-)-terremutin in infection using non-producing isolate/mutants and no clear report on the ability of these phytotoxins to produce typical *Botryosphaeria* foliar symptoms. To study this role of these phytotoxins in infection, we compared the pathogenicity on grapevine of two *N. parvum* isolates differing strongly in their (-)-terremutin and (*R*)-mellein in vitro productions. In addition, we obtained a phytotoxin biosynthetic UV mutant from a wild-type isolate producing both (-)-terremutin and (*R*)-mellein. This mutant does not produce (-)-terremutin anymore but still produced (*R*)-mellein. The pathogenicity of this mutant was compared to its parent. The genomes of these two isolates and of this mutant were sequenced to identify polymorphisms in genes involved in (-)-terremutin and (*R*)-mellein biosynthesis. We also analyzed grapevine immunity upon pathogen inoculation or after plantlet exposure to exogenous (-)-terremutin or (*R*)-mellein. Our results showed to which extent (*R*)-mellein and (-)-terremutin are necessary for the full pathogenicity of *N. parvum* on grapevine.

## 2. Material and Methods

### 2.1. Plant Material and Growth Conditions

Rooted cuttings of grapevine (*Vitis vinifera* L. cv. Chardonnay) were produced in a controlled growth room (25 °C day/night, 60% relative humidity, and 16 h photoperiod at 400 µmoles/m^2^/s) from three-node-long green stems as described by Lebon et al. [45]. Assays were repeated five times, with at least 10 cuttings per treatment. 

Plantlets of grapevine (clone 7535) were produced as described by Trotel-Aziz et al. [46]. Assays were repeated five times, with at least 3 plantlets per treatment. 

*Arabidopsis* plantlets were grown according to Millet et al. [47] with minor modifications. Briefly, bleach-sterilized seeds were placed during 10 days in 1 mL MS media with 1% sucrose in a 12 well plate. Before treatment, the growing media was refreshed. Assays were repeated three times, with at least 10 to 15 plantlets per treatment. 

### 2.2. Fungal Strains and Growth

*Neofusicoccum parvum* strains *Np*-Bourgogne S-116 (namely, Np-B) and Np-Bt67, isolated respectively from French (Bourgogne area) and Portuguese (Estremadura area) vineyards, were both registered in fungal collections from the Institut Français de la Vigne—IFV (Rhône-Méditerranée, France) and the Instituto Superior de Agronomia—ISA (Lisbon University, Lisboa, Portugal) [10,23]. Fungi were grown on potato dextrose agar medium (PDA, Sigma, St Quentin Fallavier, France) in the dark at 22 °C for 7 days before inoculating cuttings. A *N. parvum* UV mutant was also obtained from a Np-B culture grown on Medium N “Vogel’s Medium” agar plates for 5 days at 25 °C [48,49]. A 3 mm Np-B plug was harvested and plated on a weak medium water agar (20%). After 4 days of growth, plates were exposed to UV light (254 nm) for 2 h at the distance of 15 cm from the UV source, followed by 12 h incubation in the dark at 28 °C. Seventeen mycelial plugs were harvested from mycelium growing edge and transferred to Medium N agar plates. After 7 days of growth in the dark at 25 °C, phytotoxins were quantified in triplicate as indicated below. Cultures displaying a reduced production of (-)-terremutin compared to wild-type Np-B were selected and purified for further screening. One stable mutant named NpB-UV9 was selected based on its lack of production of (-)-terremutin. 

### 2.3. Production and Quantification of Phytotoxins

Once purified by Abou-Mansour et al. [23] from a 10-day-old cultures of *N. parvum* Np-B, (-)-terremutin and (*R*)-mellein were maintained as stock solutions (30 mg/L in sterile MS or MgSO_4_ 10 mM) in the dark at 4 °C, before applying to cuttings or plantlets. Phytotoxin concentrations were checked by HPLC before each application as reported by Trotel-Aziz et al. [18].

Phytotoxins production was also quantified using in vitro fungal cultures after 7, 14, and 28 days of growth on PDA in the dark at 25 °C. The whole contents of a plate (agar and mycelium) were suspended in 25 mL of methanol (HPLC grade) for an overnight continuous shaking in the dark at room temperature. The homogenate was then centrifuged at 5000× *g* for 15 min at 4 °C, and the clean supernatant was used for phytotoxin analysis by HPLC [18].

### 2.4. Fungal Inoculation of Cuttings and Disease

Green stem of 12-week-old cuttings were wounded (5 mm diameter, 1 mm deep) then inoculated with a plug from a 7-day-old PDA culture of a *N. parvum* strain as described by Trotel-Aziz et al. [18]. Controls were inoculated with PDA plugs without mycelium, and then, cuttings were maintained in the same culture chamber conditions. Symptoms of *Botryosphaeria* dieback were evaluated 4 months post-inoculation by measuring the canker area, the length of necrosis on green shoots, and the percentage of dead branches per treatment as described by Laveau et al. [50], Espinosa et al. [51], Trotel-Aziz et al. [18] and Leal et al. [15]. 

### 2.5. Plant Treatments with Phytotoxins

(-)-Terremutin was inoculated into wounded grapevine cuttings as described for *N. parvum* in 2.4. In addition, 250 µL of pure (-)-terremutin (30 mg/L in distilled water) was added to the wounded area using a hydrophilic cotton and sealed with parafilm. Four days after inoculation, leaf samples were collected and stored at −80 °C (1 g FW in 5 mL of methanol—LC-MS quality, Merck, France—for 1 h at 37 °C) before analysis of (-)-terremutin [18]. 

Eight-week-old grapevine plantlets were also treated with the phytotoxins (*R*)-mellein (350 µg/L) or (-)-terremutin (750 µg/L) in sterile MS for 72 h under the phytotronic conditions indicated by Trotel-Aziz et al. [18]. Controls corresponded to plantlets transferred to a sterile MS medium. 

*Arabidopsis thaliana* seedlings were submitted to treatments with increasing doses of (-)-terremutin (0.1 µM to 100 µM). Additionally, a set of *A. thaliana* seedlings (10–15) was treated with 200 µM MeJA and 100 µM (-)-terremutin simultaneously. 

### 2.6. Plant RNA Extraction and qRT-PCR Analysis

Samples from cuttings (pool of 20 leaves from 10 plants per treatment) and plantlets (pool of three shoots per treatment) were collected respectively at 4 days after inoculation of the cuttings with *N. parvum* or at 3 days after treatment of the plantlets with phytotoxins, ground in liquid nitrogen, and then stored at −80 °C. Total RNA was extracted from 50 mg of leaf powder for cuttings or from 100 mg of plantlet shoots powder with PlantRNA Purification Kit as described by Trotel-Aziz et al. [18]. Then RNA quality was assessed, total RNA concentration measured, first-strand cDNA was synthesized, and quantitative RT-PCR (qRT-PCR) was performed [18] with Absolute Blue qPCR SYBR Green ROX Mix according to manufacturer instructions (Thermo Fisher Scientific, Inc., Waltham, MA, United States), in a BioRad C1000 thermocycler using the BioRad manager software CFX96 Real Time PCR (BioRad, Hercules, CA, United States). The expression of 13 grapevine defense genes, selected for their response to pathogens or their virulent factors [13,18,29,52], was monitored by qRT-PCR using the primers shown in Table S3. Analyses of gene expression by qRT-PCR were repeated five-times from independent experiments. Results of gene expression correspond to means ± standard deviation from one representative out of at least three showing the same trends. Changes in gene expression levels were considered significant when their expression differed from the control sample by >2-fold or <0.5-fold, respectively. Control cuttings were inoculated with sterile PDA plugs (expression level set at 1), while control plantlets grew on MS medium (expression level set at 1). For *Arabidopsis* seedlings, the extraction protocol from 100 mg of shoot powder from three plantlets was similar with some modifications. Trizol was used as an extraction reagent, final cDNA concentration was adjusted to 500 ng/µL and reverse transcription was performed with 500 µg of cDNA. The *AtVSP* gene was chosen to test JA responsiveness as described in Van Wees et al. [53].

### 2.7. Fungal DNA Extractions and Genome Sequencing

Mycelium from 7-days old *N. parvum* PDA cultures was recovered by scraping the agar plate with a sterile scalpel blade and stored at −80 °C until use. Samples were then lyophilized and ground to fine powder with liquid nitrogen. Genomic DNA was extracted according to Mayjonade et al. [54] for NGS Illumina sequencing. High-throughput paired-end sequencing (2 × 150 bp) of Np-B, NpB-UV9 and Np-Bt67 genomes was performed by Genewiz (GENEWIZ, Inc., South Plainfield, NJ, USA), using the Illumina HiSeq platform. Sequence data quality was assessed for each sample with FastQC 0.10.1 [55]. Low quality bases were trimmed using Trimmomatic 0.36 [56], with the following parameters: LEADING:20 TRAILING:20 SLIDINGWINDOW:4:15 MINLEN:50 HEADCROP:2. The reads were mapped to the Np-UCR-NP2-v3 *N. parvum* reference genome sequence [57] using BWA-mem 0.7.16 [58] as defaults parameters. Sorting and indexing of the aligned sequences were performed with Samtools 1.4 [59]. Picard 2.2.4 software (http://broadinstitute.github.io/picard/, accessed on 1 March 2018) was used to remove duplicates and Bamaddrg (https://github.com/ekg/bamaddrg, accessed on 1 March 2018) to edit read groups information. Coverage was evaluated using Bedtools 2.17.0 [60]. 

### 2.8. Genome Bioinformatic Analysis

Variant calling from alignment files was performed with Freebayes 1.1.0-60-gc15b070 [61], with the ploidy parameter set to 1. SnpSift 4.3t [62] was used to detect variants differing between each pair of samples, and to filter out variants not supported by at least 5 reads. To reduce false positives linked to mapping ambiguities, we also filtered out variants according to their allelic frequencies with an in-house script, keeping only variants with a major allele frequency >0.8. Annotation of variants was achieved with SnpEff [63]. The phylogenetic tree was constructed using default parameters at phylogeny.fr [64], and concatenated sequences of housekeeping genes actin, alpha-tubulin, beta-tubulin, calmodulin, EF1, EF2, EF3, RPB1 and RPB2 of all studied isolates (Figure S2). To detect genes involved in (-)-terremutin and (*R*)-mellein synthesis, the genome sequence of the reference isolate Np-UCR-NP2-v3 was analyzed with AntiSMASH fungal version, with defaults parameters [65]. 

### 2.9. RNAseq Gene Expression Data Analysis and Gene Re-Annotation

A public RNAseq dataset (GSE85079) obtained with *N. parvum* isolate Np-UCD-646-So [36] was used to improve Np-UCR-NP2-v3 gene annotation and to assess gene expression of mellein synthase and terremutin cluster genes. RNAseq reads from 3 PDA-grown samples were mapped against Np-UCR-NP2-v3 genome using HISAT2 v2.2.0 tool [66].

### 2.10. Statistical Analysis

Experiments with grapevine cuttings and plantlets were repeated five times. All data are means ± standard deviations. Statistical analyses were carried out using the SigmaStat 3.5 software and mean values were compared by Tukey’s test (*p* < 0.05).

## 3. Results

### 3.1. Pathogenicity on Grapevine of N. parvum Isolates Differing in (-)-Terremutin and (R)-Mellein In Vitro Production

**Figure 1:**
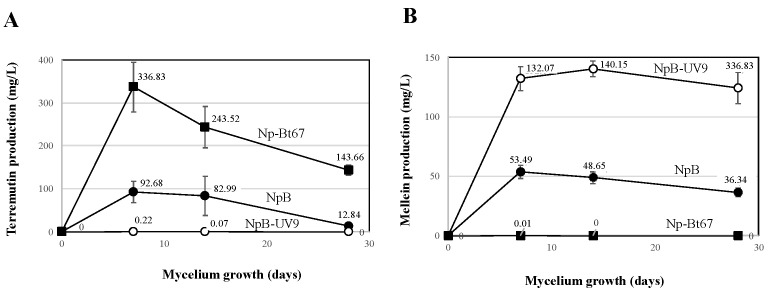
(-)-Terremutin and (*R*)-mellein in vitro production by *N. parvum* (Np). Isolates Np-B, NpB-UV9, and Np-Bt67 were grown on potato dextrose agar for one month. (-)-Terremutin (**A**) and (*R*)-mellein (**B**) were extracted from culture medium and quantified according to Trotel-Aziz et al. [18] using an HPLC coupled to a diode array detector.

*N. parvum* (Np) strains were analyzed for their (-)-terremutin and (*R*)-mellein in vitro production (Figure 1). *N. parvum*-Bt67 isolate (Np-Bt67) produced high amounts of (-)-terremutin (336 mg/L) but no (*R*)-mellein (0.01 mg/L), while *N. parvum*-Bourgogne S-116 isolate (Np-B) produced significant amounts of both (-)-terremutin and (*R*)-mellein (92 and 53 mg/L respectively, Figure 1). In contrast, the NpB-UV9 mutant derived from Np-B by UV mutagenesis (NpB-UV9) produced very low amounts of (-)-terremutin (0.2 mg/L, 421-fold decrease) and overproduced (*R*)-mellein by 2.5-fold (132 mg/L, Figure 1). 

**Figure 2:**
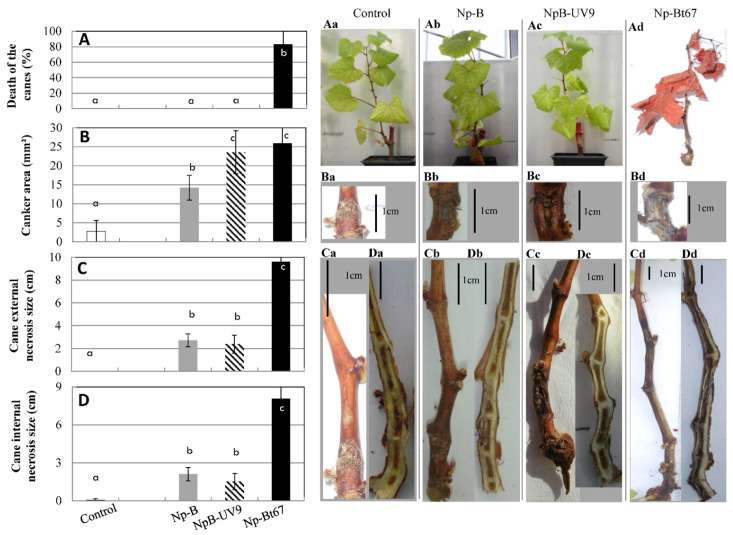
Pathogenicity of *N. parvum* isolates Np-B, NpB-UV9, or Np-Bt67 on grapevine. *N. parvum* strains Np-B, NpB-UV9, and Np-Bt67 were inoculated to wounded three-months Chardonnay grapevine cuttings using mycelial plugs. Non-infected plants were inoculated with plugs from the sterile medium (Control). Typical Botryosphaeria dieback symptoms were monitored during 4 months: dead branch (**A**), stem canker (**B**), stem external necrosis (**C**), and stem internal necrosis (**D**). Photos of corresponding phenotypes, pathogen-free (a) or infected with Np-B (b), NpB-UV9 (c) or Np-Bt67 (d), show cane death (**Aa**–**Ad**), canker (**Ba**–**Bd**), and external and internal necrosis (**Ca**–**Cd**,**Da**–**Dd**), respectively. Data are means ± standard deviation (SD) for at least three independent experiments with ten biological replicates by treatment. Vertical bars sharing different letters are statistically significantly different (Multiple Comparison procedures with Tukey’s test, *p* < 0.05).

The pathogenicity on grapevine of these isolates was tested by inoculating wounded Chardonnay cuttings. At 4 mpi, Np-Bt67 induced *Botryosphaeria* dieback symptoms on canes both externally (canker, necrosis) and internally (necrosis) (Figure 2). In addition, 83% of the Np-Bt67 infected grapevine cuttings displayed dead canes (Figure 2A,Ad). Np-Bt67 induced external cankers with an average surface of 26 mm^2^ (Figure 2B,Bd) and external- and internal- necrosis with an average size of 9.6 and 8.1 cm, respectively (Figure 2C,D,Cd,Dd). Np-B was less aggressive on grapevine cuttings than Np-Bt67. No dead cane was observed following Np-B inoculation (Figure 2A,Ab). Np-B induced cane necrosis with an average size significantly lower in sizes than those observed for Np-Bt67 both externally (2.7 cm, 3.5-fold decrease, Figure 2C,Cb) and internally (2.1 cm, 3.8-fold decrease, Figure 2D,Db). These differences were statistically highly significant. Canker sizes induced by Np-B were also lower in surface than those induced by Np-Bt67 (14.3 mm^2^, 1.8-fold, Figure 2B,Bb), and these differences were statistically significant. The pathogenicity of NpB-UV9 to grapevine cuttings was similar to Np-B concerning dead cane percentage and the average size of internal and external cane necrosis induced by the infection (Figure 2A,C,D,Ac,Cc,Dc). However, NpB-UV9 induced bigger cankers than Np-B (23.5 mm^2^, 1.6-fold increase, Figure 2B,2Bc), and these differences were statistically significant. 

### 3.2. Effect of (-)-Terremutin on the Pathogenicity of N. parvum Isolates

**Figure 3:**
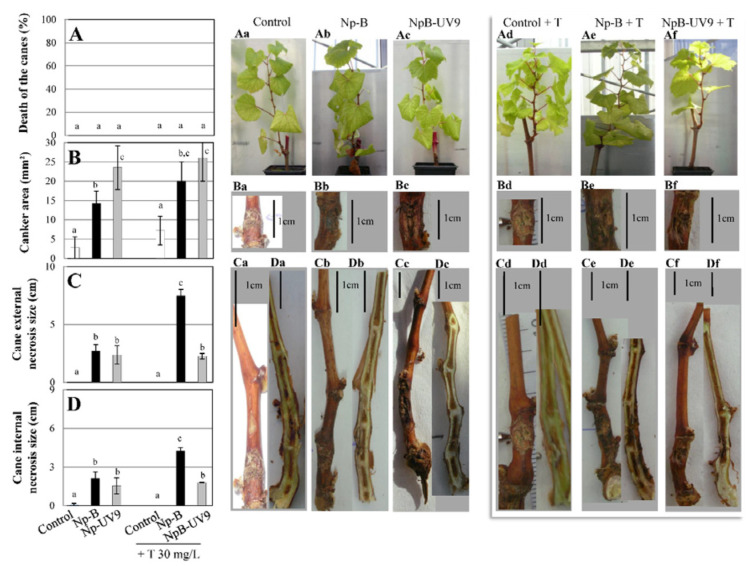
Effect of (-)-terremutin on *N. parvum* pathogenicity on grapevine. Three months old Chardonnay grapevine cuttings were co-inoculated with (-)-terremutin (30 mg/L) and *N. parvum* isolates Np-B and NpB-UV9 as described in Figure 2. Non-infected plants were inoculated with a plug sterile medium (Control). Typical Botryosphaeria dieback symptoms were monitored during 4 months: dead branch (**A**), stem canker (**B**), stem external necrosis (**C**), and stem internal necrosis (**D**). Photos of corresponding phenotypes: pathogen-free (a), infected with Np-B (b), infected with NpB-UV9 (c), pathogen-free + (-)-terremutin (d), co-infected with Np-B + (-)-terremutin (e) or NpB-UV9 + (-)-terremutin (f), show cane death (**Aa**–**Af**), canker (**Ba**–**Bf**), and external and internal necrosis (**Ca**–**Cf**,**Da**–**Df**), respectively. Data are means ± standard deviation (SD) for at least three independent experiments with ten biological replicates by treatment. Vertical bars sharing different letters are statistically significantly different (Multiple Comparison procedures with Tukey’s test, *p* < 0.05).

The addition of 30 mg/L (-)-terremutin alone did not induce foliar symptoms on grapevine cuttings (Figure 3Ad) compared to control cuttings (Figure 3Aa), and neither cane necrosis (Figure 2Ca,Cd and Figure 3C,D,Da,Dd) nor dead canes (Figure 3A,Aa,Ad). In addition, the average surface of cankers induced by (-)-terremutin alone was higher (7.3 mm^2^; 2.6-fold; Figure 2B,Ba,Bd) than those induced only by wounding, although these differences were not statistically significant.

Exogenous (-)-terremutin was also added to infected grapevine cuttings, at the same time and in the same place as the mycelium plug containing the *N. parvum* isolate Np-B or its UV mutant NpB-UV9 that do not produce (-)-terremutin in vitro. The addition of 30 mg/L (-)-terremutin to Np-B increased canker area by 1.4-fold (20 mm^2^; Figure 3B,Bb,Be) and increased significantly both cane external necrosis length by 2.8-fold (7.5 cm; Figure 3C,Cb,Ce) and cane internal length by 2-fold (4.3 cm; Figure 3D,Db,De). However, these sizes of cane necrosis induced by this treatment, were smaller than those observed for the strong (-)-terremutin-producing isolate Np-Bt67 (9.6 cm and 8.1 cm respectively, Figure 2C,D). Treatment with (-)-terremutin did not affect the pathogenicity of the UV mutant NpB-UV9 (Figure 3Af–Df) compared to its parent Np-B (Figure 3Ae–De).

### 3.3. N. parvum and Its Phytotoxins (R)-Mellein and (-)-Terremutin Interfere with Grapevine Defense Gene Expression

**Figure 4:**
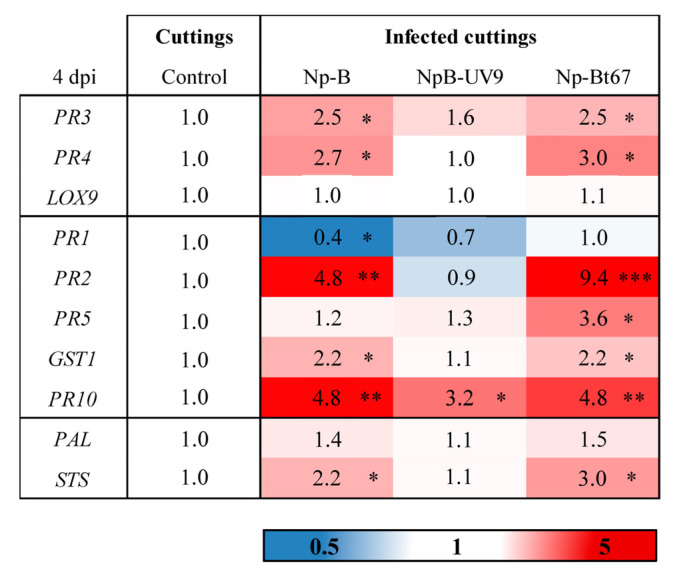
Effect of *N. parvum* infection on the expression of grapevine defense genes. Grapevine cuttings were inoculated with the *N. parvum* isolates Np-B, NpB-UV9, and Np-Bt67 as described in Figure 2. Non-infected plants were inoculated with a plug sterile medium (Control). Transcript levels of grapevine defense-related genes were quantified in leaves from infected grapevine cuttings by RT-qPCR 4 days after infection (dpi). Results are from one representative replicate among three independent experiments. The fold induction values were normalized to the expression of the reference genes *EF1* and *60SRP* as internal controls and to Control cuttings (expression level assigned to 1 for each gene). A three colors scale was used to show the expression level of each gene. Red shades indicate an overexpression and deep red corresponds to an induction factor of 5 or more; white is the basal expression level that does not differ from the Control; blue shades indicate a repression and dark blue corresponds to a 0.5-fold induction or less. Different asterisks indicate significant differences. *PR3* = acidic class IV chitinase; *PR4* = pathogenesis-related protein 4; *LOX9* = lipoxygenase 9; *PR1* = pathogenesis-related protein 1; *PR2* = β-1,3-glucanase; *PR5* = pathogenesis-related protein 5; *GST1* = glutathione-S-transferase 1; *PR10* = pathogenesis-related protein 10; *PAL* = phenylalanine ammonia-lyase; *STS* = stilbene synthase.

A set of grapevine defense genes was selected based on our previous studies on the induction of grapevine defenses upon infection by *N. parvum* [13,18,52,67]. At 4 dpi, the expression of three SA-responsive genes (*PR2*, *PR5*, *PR10*) was induced in leaves of grapevine cuttings infected with *N. parvum* isolates (Figure 4). Upon infection with the most aggressive isolate Np-Bt67, the induction of these three SA-responsive genes was increased from 3.6 to 9.4 compared to the control, and 1.5 to 10.4-fold higher than upon challenge with Np-B or NpB-UV9. Interestingly, the less aggressive isolate Np-B significantly repressed the expression of the SA-responsive gene *PR1* (0.4-fold) compared to mock. The JA-responsive gene *LOX9* was not up-regulated upon infection with the different Np strains, while the expression of the JA/ET-responsive gene *PR3* encoding a chitinase was increased by 2.5-fold upon Np-Bt67 or Np-B infection, and by 1.6-fold upon NpB-UV9 infection (Figure 4). The expression of the JA/ET-responsive gene *PR4* encoding a pathogenesis-related protein 4 was also increased upon Np-Bt67 or Np-B infection, by 3.0-fold and 2.7-fold respectively (Figure 4). The expression of three other genes involved in the synthesis of antimicrobial phytoalexins (*PAL* and *STS*) and in the reactive oxygen species detoxification (*GST1*) was also assessed. The expression of these three genes was higher in cuttings infected by Np-Bt67 and Np-B than in cutting infected by NpB-UV9 (Figure 4).

**Figure 5:**
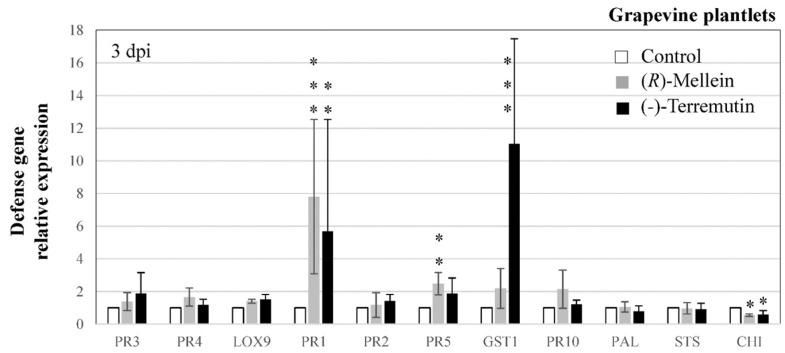
Effect of (*R*)-mellein and (-)-terremutin on the expression of grapevine defense genes. Grapevine plantlets were transferred in a MS medium containing either (-)-terremutin 750 µg/L, (*R*)-mellein 350 µg/L or no phytotoxin (Control). Transcript levels of grapevine defense-related genes were quantified by RT-qPCR in plantlets three days after treatment. The fold induction values were normalized to the reference genes *EF1* and *60SRP* as internal controls and to Control plantlets (expression level assigned to 1 for each target gene). Results are from one representative replicate among five independent experiments showing the same trends. Different asterisks indicate significant differences. Legends as in Figure 3. *CHI* = chalcone isomerase.

We also fed grapevine plantlets with (*R*)-mellein and (-)-terremutin and monitored changes in host defense responses at 3 dpi (Figure 5). The expressions of *PR1*, *PR5* and *GST1* were up-regulated by one or both phytotoxins, from 2.48 to 11-folds depending on the phytotoxin (*PR1* from 5.2 to 7.8, *PR5* from 1.86 to 2.48, and *GST1* from 2.19 to 11.04, in response to (R)-mellein or (-)-terremutin, respectively), whereas that of *CHI* was severely down-regulated by both phytotoxins (0.5-fold change, Figure 5). 

Grapevine defense genes expression levels from *N. parvum* infected cuttings were compared to those of plantlets treated with phytotoxins (Figure 4 and Figure 5). *PR1* was downregulated in infected cuttings (0.4-fold), while it was up-regulated by both phytotoxins (5.2 to 7.8-fold). On the other hand, JA/ET-responsive genes were less up-regulated in plantlets treated with phytotoxins (up to 1.87 and 1.66, respectively for *PR3* and *PR4*) than in infected cuttings (2.5 and 3.0-fold, respectively). *PR2* and *PR10* were also less up-regulated in plantlets treated with phytotoxins (up to 1.43 and 2.15, respectively) than in infected cuttings (up to 9.4 and 4.8-fold, respectively), and similarly for *STS* (up to 0.97-fold in plantlets facing phytotoxins, and up to 3.0-fold in infected cuttings). Finally, *PR5* and *GST1* were both over-expressed in response to pathogen infection and phytotoxin treatments, especially with (-)-terremutin and with the most aggressive isolate Np-Bt67 that overproduces (-)-terremutin. 

To assay if (-)-terremutin interferes with JA-dependent defense responses, as the phytotoxin most commonly produced by the most aggressive pathogen, we treated *Arabidopsis* plantlets with (-)-terremutin and JA. Compared to the positive control (MeJA), (-)-terremutin alone did not induce the expression of *AtVSP* (Figure S1), whereas the simultaneous supply of MeJA and (-)-terremutin reduced *AtVSP* expression, supporting the hypothesis that (-)-terremutin interferes with the plant JA-dependent defense responses. 

### 3.4. Identification of (-)-Terremutin and (R)-Mellein Biosynthetic Genes in the Genome Sequence of N. parvum

**Figure 6:**
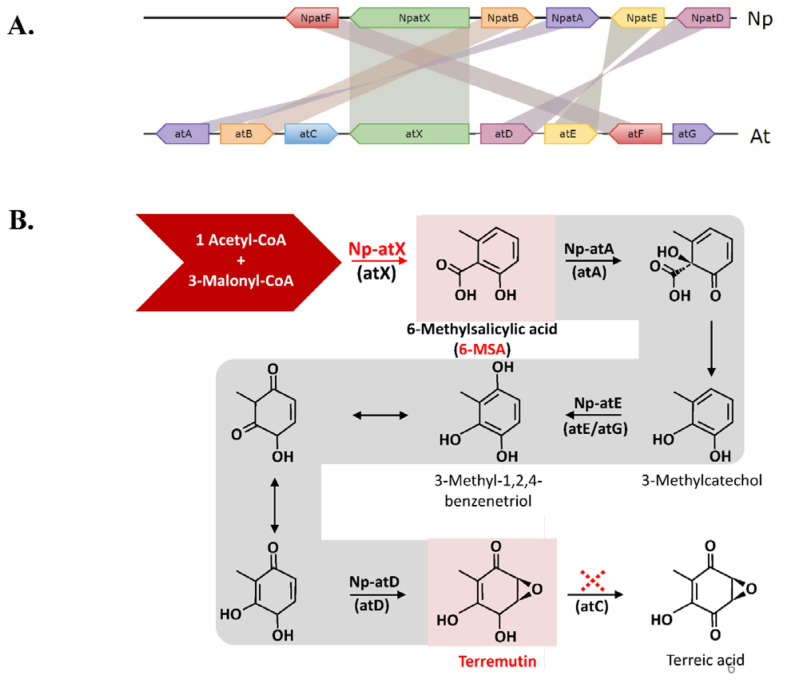
*N. parvum* terremutin biosynthesis gene cluster. The genome sequence of *N. parvum* isolate Np-UCR-NP2-v3 [57] was analyzed with Antismash [36]. The contig KB916132.1 carried five genes encoding proteins with similarities to those involved in the biosynthesis of terremutin/terreic acid in *A. terreus* [33,34], named *Np-atF, Np-atX, Np-atB, Np-atA,* and *Np-atE/D* (**A**). There is no ortholog of *atC* from *A. terreus* in *N. parvum* genome. *A. terreus atC* encodes an GMC oxidoreductase that catalyzes the conversion of terremutin into terreic acid (**B**). Therefore, the organization of *N. parvum* terremutin biosynthetic gene cluster (lack of *atC* ortholog) is in agreement with the observation that this fungal species produce only (-)-terremutin, but not terreic acid.

**Figure 7:**
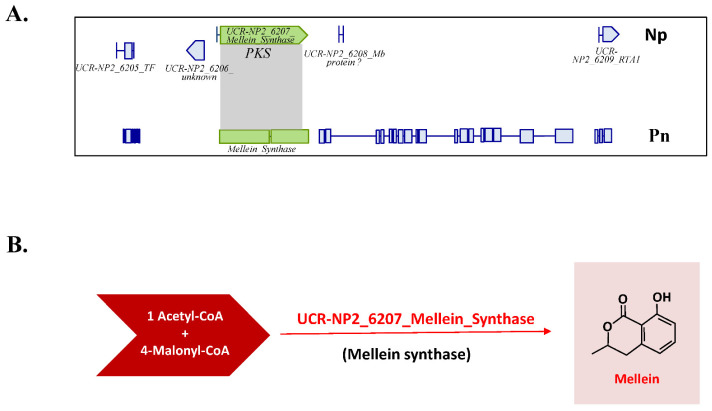
*N. parvum* genomic locus for mellein synthase gene. The genome sequence of *N. parvum* isolate Np-UCR-NP2-v3 [57] was analyzed with Antismash [36]. The contig (KB916367.1) carries a gene (Np-UCR-NP2_6207.2) encoding a PKS with 95% similarities with the mellein synthase from *P. nodorum* (**A**), [31]. The biosynthetic pathway for mellein is outlined in (**B**).

The available genome sequence of *N. parvum* isolate Np-UCR-NP2-v3 [57] was analyzed with Antismash [65] to detect genes involved in secondary metabolism (SM). Overall, 60 SM biosynthetic gene clusters (BGCs) were detected in the genome of Np-UCR-NP2-v3 (Table S1). Ten BGCs carried out a single gene encoding a NRPS, ten a NRPS-like, 18 a PKS, and 11 a terpene synthase, while other BGCs carried out combinations of NRPS, PKS, hybrid PKS-NRPS, and terpene synthase. Among these 60 BGCs, 11 have significant similarities (>30%, protein level, Table S1) with SM gene clusters from other fungi involved in the biosynthesis of different secondary metabolites such as dimethylcoprogen, DHN melanin, patulin, alternapyrone, pyranonigrin, ilicicolin, mellein and terremutin/terreic acid. The *N. parvum* genome sequence contig KB916132.1 carried five genes encoding proteins with similarities to those involved in the biosynthesis of terremutin/terreic acid in *A. terreus* (atA, atB, atD, atE, atF, and atX, Figure 6A) that were named *Np-atF, Np-atX, Np-atB, Np-atA* and *Np-atE/D* [33,34]. The structure of these *N. parvum* genes was manually refined using RNAseq data from *N. parvum* isolate Np-UCD-646-So [36], and protein sequences generated by Antismash (Table S4). We noticed that the Np-UCR-NP2-v3 annotation of *Np-atE/D* corresponded to the fusion of two distinct closely located genes. As a consequence, Np-atE/D was renamed as *Np-atE* (Np-UCR-NP2_4359.1), and *Np-atD* (Np-UCR-NP2_4359.2) as indicated in Figure 6. This novel version of Np-atD protein sequence was almost similar (99 % identity) to a protein sequence from the genome annotation of *N. parvum* isolate Np-UCD-646-So (NpatD_Np-UCD-646-So_9532, [36]). The structure of *Np-atB* was also modified (Np-UCR-NP2_4357) according to RNAseq data (Table S4). Overall, we identified six of the eight genes from *A. terreus* terremutin/terreic acid cluster in *N. parvum* genome organized as a cluster (Figure 6, Table S4), including the PKS *Np-atX* highly similar (94 %) to the PKS from *A. terreus* encoded by *atX* (Figure S2). The two genes *atG* and *atC* from *A. terreus* terremutin/terreic acid cluster are missing in *N. parvum* genome (Figure 6). *atG* encodes a P450 cytochrome oxidase which does not seem to have an essential role in *A. terreus* terremutin/terreic acid biosynthesis, as it may be replaced by P450 cytochrome oxidase encoded by *atE* [33,34]. *atC* encodes an GMC oxidoreductase that catalyzes the conversion of terremutin into terreic acid [33,34]. The *A. terreus atC* deletion mutant was obtained and it accumulated terremutin instead of terreic acid [33]. The organization of the *N. parvum* terremutin gene cluster suggests that this fungal species will produce (-)-terremutin but not terreic acid (Figure 6). This is indeed the case as (-)-terremutin was detected in culture filtrates of the *N. parvum* isolates Np-Bt67 and Np-B, but not terreic acid (Figure 1). The search for genes involved in mellein biosynthesis in *N. parvum* reference genome sequence Np-UCR-NP2-v3 [57] highlighted a contig (KB916367.1) carrying a gene (Np-UCR-NP2_6207.2) encoding a PKS with 95% similarities with the mellein synthase from *P. nodorum* (Table S1, Figure 7, [31]).

### 3.5. Genetic Diversity of N. parvum Genes Involved in (-)-Terremutin and Mellein Biosynthesis

To identify genomic traits associated with differences observed in (-)-terremutin and mellein production between *N. parvum* isolates, we sequenced the genomes of the two isolates Np-Bt67 and Np-B using Illumina NGS. We first aligned the sequencing reads of these two isolates against the *N. parvum* reference genome from isolate Np-UCR-NP2 [57]. Variants differing between isolates were extracted from alignments and filtered for their quality. A phylogenetic tree was constructed using housekeeping genes sequences (*actin*, *alpha-tubulin*, *beta-tubulin*, *calmodulin*, *EF1*, *EF2*, *EF3*, *RPB1* and *RPB2*) from all studied strains (Figure S3). This phylogenetic analysis showed that Np-Bt67 was more closely related to Np-UCR-NP2 than to Np-B, and that Np-B was closely related to Np-UCD-646-So. All genes identified in Np-UCR-NP2 genome sequence as involved in (-)-terremutin and mellein biosynthesis (Figure 6 and Figure 7, Table S2) were also detected in Np-Bt67 and Np-B without early stop codon or frameshift mutations that could impair (-)-terremutin or mellein biosynthesis. Nine nucleotide differences leading to an amino acid change (Table S2, highlighted lines), were identified in five of the six genes from *N. parvum* (-)-terremutin cluster: Np-atF (transcription factor: G723, E743D), Np-atX (PKS: D471G, S957N, A1237T, A1248T), Np-atB (MFS: E11D), Np-atE (P450: T31I), and Np-atD (epoxidase: D55E). Eleven nucleotide differences leading to an amino acid change, were identified in *N. parvum* gene Np-UCR-NP2_6207 encoding the PKS mellein synthase (Table S2, highlighted lines). We also sequenced the genome of the UV mutant NpB-UV9 to identify genomic traits that could explain its highly reduced (-)-terremutin production compared to its parent Np-B (Figure 1). The genome sequences of these two strains were compared using *N. parvum* genome sequence of isolate Np-UCR-NP2-v3 [57] as a cross-reference. Differences for fourteen nucleotides were detected between Np-B and NpB-UV9, but only two were located in coding sequences (Table 1). Each mutation affected a distinct gene encoding either a putative Zn(2)-Cys6 transcription factor conserved in fungi (Np-UCR-NP2_6692), or a putative regulator of G protein signaling conserved in fungi highly similar (78%) to the flbA protein of *Aspergillus nidulans* [68] involved in sporulation (Np-UCR-NP2_9007). Both genes were re-annotated using available RNAseq data (Table S4). Newly annotated versions of genes UCR-NP2_6692 and UCR-NP2_9007 were named UCR-NP2_6692_v2 and UCR-NP2_9007-v2 respectively. Np-UCR-NP2_6692 mutation affected the protein sequence (P603A), while Np-UCR-NP2_9007 mutation was located in an intron and is likely silent (Table 1). 

## 4. Discussion

Several phytotoxic secondary metabolites produced by *N. parvum* have been identified, but their roles in the infection of grapevine and in the production of foliar *Botryosphaeria* dieback symptoms are still unclear [17,18,21,23,24,25,26,27]. To evaluate the role in grapevine infection of the *N. parvum* phytotoxins (-)-terremutin and (*R*)-mellein, we compared the pathogenicity of two natural isolates and one mutant differing in the in vitro production of these phytotoxins. Wild-type strains of *N. parvum* Np-B and Np-Bt67 differed in their in vitro production of (-)-terremutin quantitatively (Np-Bt67 > 3-fold Np-B) and of (*R*)-mellein qualitatively (Np-Bt67 is a mellein almost non-producer, Figure 1). We also obtained a UV mutant (NpB-UV9) differing from its parent Np-B by its inability to still produce (-)-terremutin (Figure 1). The pathogenicity on grapevine of these isolates/mutant was assessed and their genome sequences were compared to highlight genetic differences associated with (-)-terremutin and (*R*)-mellein production. 

### 4.1. Role of (-)-Terremutin in the Pathogeniciy of N. parvum on Grapevine

Np-Bt67 induced severe apoplectic form of *Botryosphaeria* dieback and strong disease symptoms characterized by (i) large cankers, (ii) dead branches, and (iii) stem necrosis as already reported for this particular *N. parvum* isolate [15,18]. Np-B and its mutant NpB-UV9 were equally pathogenic, but less pathogenic to grapevine than Np-Bt67 (no dead branches, no apoplectic foliar symptoms, reduced stem necrosis, reduced cankers). Np-Bt67 was therefore the only isolate to induce severe damages (i.e., dead branches) and apoplexy within 15 days. The secondary metabolite profile of Np-Bt67 was characterized by its ability to produce high amount of (-)-terremutin (3-fold higher than Np-B, Figure 1), but almost no (*R*)-mellein. We hypothesized that the high production of (-)-terremutin by Np-Bt67 was responsible for its high level of aggressiveness on grapevine and its ability to induce the collapse of branches and foliar symptoms. Further experiments were carried out to confirm or disprove this hypothesis, which is consistent with the literature describing (-)-terremutin as an abundant cyclohexenoid that induces the greatest extent of necrosis on grapevine leaf discs [17]. First, (-)-terremutin supply to both control and infected grapevine cuttings did not induce any branches death, nor foliar symptoms, but it induced a local necrosis leading to small cankers (Figure 2). This strongly suggests that (-)-terremutin is not the main factor involved in the ability of Np-Bt67 to induce dead branches and foliar symptoms. Second, the lower (-)-terremutin producer wild-type isolate Np-B (3 fold less than Np-Bt67) was co-inoculated on grapevine cuttings with purified (-)-terremutin (Figure 2). Although this co-inoculation did not induce dead branches, the sizes of internal/external stem necrosis were increased from 2- to 3-fold by the addition of (-)-terremutin. Thus, even if (-)-terremutin is not sufficient alone to induce dead branches, it increased the aggressiveness of the lower (-)-terremutin producer Np-B. Third, we compared the pathogenicity of the isolate Np-B to its mutant NpB-UV9 unable to produce (-)-terremutin. Both strains displayed the same level of aggressiveness to grapevine, strongly suggesting that (-)-terremutin is not required for pathogenicity on grapevine. The addition of (-)-terremutin to mutant NpB-UV9 did not increase its ability to induce stem necrosis, as observed with the (-)-terremutin producer Np-B. This unexpected result could be explained by the fact that exogenous (-)-terremutin is not provided in sufficient amount to increase the aggressiveness of NpB-UV9, but in sufficient amount when added to the (-)-terremutin producing isolate Np-B. Overall, our results suggest that (-)-terremutin acts more as a virulence factor (i.e., increasing quantitatively the disease) than as a pathogenicity factor (i.e., required to trigger the disease) upon infection [18,28,31]. Indeed, the review by Masi et al. [17] on the role of fungal phytotoxins in the development of GTDs clearly highlights (1) indirect evidence relying phytotoxins to grapevine diseases and (2) the need for further studies to remove any doubt about the actual involvement of phytotoxins in pathogen virulence. To demonstrate our hypothesis, (-)-terremutin non-producing mutants should be obtained by targeted gene replacement of the polyketide synthase Np-atX gene in both Np-B and Np-Bt67, and their pathogenicity on grapevine should be evaluated in comparison to their wild-type parent. 

### 4.2. Role of (R)-Mellein in the Pathogenicity of N. parvum on Grapevine

Np-Bt67 secondary metabolite profile was characterized by its inability to produce (*R*)-mellein, while Np-B and NpB-UV9 both produced large amounts of (*R*)-mellein in culture (53–140 mg/L, Figure 1). The fact that the (*R*)-mellein non-producing isolate Np-Bt67 was the most pathogenic isolate on grapevine strongly suggests that (*R*)-mellein is not required for pathogenicity. These results are similar to those obtained by Chooi et al. [31] in the wheat pathogenic fungus *Parastagonospora nodorum*. These authors showed that a *P. nodorum* mutant unable to produce (*R*)-mellein was as pathogenic on wheat as its wild-type parent. 

NpB-UV9 produces 2.5-fold more (*R*)-mellein in culture than its parent Np-B. Since NpB-UV9 is as pathogenic as Np-B, except for the size of the cankers induced by the infection that are 1.6-fold bigger for NpB-UV9 than for Np-B (Figure 2), these results suggest that the ability of NpB-UV9 to produce high levels of (*R*)-mellein is associated with the induction of larger external cankers. However, Np-Bt67 that failed to produce (*R*)-mellein was able to induce cankers of the same size as NpB-UV9, suggesting that this hypothesis is difficult to support, unless (*R*)-mellein levels strongly differs between in vitro and *in planta* production, as suggested by Reveglia et al. [30]. Indeed, only few quantification studies have performed in infected plant tissues as these metabolites are difficult to detect [17]. Overall, our results suggest that (*R*)-mellein acts more as a virulence factor (i.e., increasing quantitatively the disease) than as a pathogenicity factor (i.e., required to trigger the disease) upon infection [18,28,31]. In good agreement with this hypothesis, (*R*)-mellein has been described as a phytotoxin with a low activity on grapevine leaf discs [17]. To demonstrate our hypothesis, (*R*)-mellein non-producing mutants should be obtained by targeted gene replacement of the mellein polyketide synthase encoding gene in both Np-B and NpB-UV9, and their pathogenicity on grapevine should be evaluated in comparison to their wild-type parent. 

### 4.3. Genetic Differences among N. parvum Isolates for Genes Involved in (-)-Terremutin and (R)-Mellein Biosynthesis

Available genome sequences of *N. parvum* isolates (Np-UCR-NP2 and Np-UCD-646-So) and Np-Bt67 and Np-B genome sequences produced in the framework of this study were used to estimate the genetic relatedness between these isolates. We also identified polymorphisms in genes involved in (-)-terremutin and (*R*)-mellein biosynthesis among these four isolates. Such high level of genetic variations within a species could be a consequence of the evolution and adaptation of *N. parvum* to its biotic environment as already suggested [69]. The phylogenetic tree constructed with Np-UCR-NP2, Np-UCD-646-So, Np-Bt67 and Np-B sequences clearly showed that Np-B/Np-UCD-646-So and Np-Bt67/Np-UCR-NP2 clustered as separate clades (Figure S3). In particular, Np-B and Np-UCD-646-So are likely closely related. All genes encoding for enzymes and proteins involved in these metabolic pathways are present in the four available *N. parvum* genome sequences (Figure 6 and Figure 7, Table S2). The only exception to this rule was the ortholog of *A. terreus AtC* encoding for a glucose-methanol-choline oxidoreductase required for the conversion of (-)-terremutin into terreic acid [32,33,34]. This gene was absent from the genomes of all *N. parvum* isolates analyzed, suggesting that *N. parvum* isolates are only able to produce (-)-terremutin, but not terreic acid (Figure 6, Table S2). Comparing the four available genome sequences identified polymorphisms in 5 of the 6 genes from the BGC involved in (-)-terremutin biosynthesis (Table S2), only few corresponded to amino acid changes between proteins of the high-producer Np-Bt67 and the medium-producer Np-B. One striking difference is the deletion of a G (G723) in the sequence of the transcription factor Np-atF of Np-Bt67 compared to allelic forms found in Np-B and Np-UCD-646-So (Table S2). The deletion of a key structural amino acid such as a glycine may have an effect on the protein. This deletion was also found in the isolate Np-UCR-NP2 related to Np-Bt67. The other differences observed between Np-Bt67 and Np-B in (-)-terremutin BGC protein sequences were limited to few amino acid changes, mostly with not major structural effect (E to D, T to I). We hypothesized that the allelic form of Np-AtF transcription factor of Np-Bt67 is more efficient than the allelic form of Np-B, explaining the high-production of (-)-terremutin by Np-Bt67. This hypothesis needs to be validated by introducing the Np-Bt67 allele of *Np-AtF* in Np-B and testing possible changes in (-)-terremutin production. Many differences were observed between the protein sequences of the mellein synthase of the almost non-producer Np-Bt67 and the high-producer Np-B (11 amino acid changes for a protein of 1779 amino acids, Tables S2 and S4). This level of genetic diversity is higher than between the PKS 6-MSA synthase *Np-atX* encoding gene of Np-Bt67 and Np-B (4 amino acid changes for a protein of 1803 amino acids, Tables S2 and S4). We hypothesized that the allelic form of Np-UCR-NP2_6207 mellein synthase from Np-Bt67 is not functional, explaining the non-production of (*R*)-mellein by this isolate in vitro. However, the structure-function relationships of these proteins have not been determined hampering any prediction of changes in function according to the protein sequence. This hypothesis needs to be validated by complementing Np-Bt67 with the Np-UCR-NP2_6207 allele of Np-B and testing possible changes in (*R*)-mellein production.

The genome sequence of the mutant NpB-UV9 was compared to its parent Np-B. Few genomic differences were identified (2 SNPs in coding regions, Table 1). One mutation was identified in a gene encoding a putative Zn(2)-Cys6 transcription factor conserved in fungi (Np-UCR-NP2_6692). The other mutation in coding sequences was identified in a gene encoding a putative regulator of G protein signaling conserved in fungi highly similar (78%) to the flbA protein of *Aspergillus nidulans* (Np-UCR-NP2_9007) [68]. Np-UCR-NP2_6692 mutation affected the protein sequence (P603A), while Np-UCR-NP2_9007 mutation was located in an intron and is likely silent. Since, we did not identify mutations in genes involved in (-)-terremutin biosynthesis between Np-B and NpB-UV9, we hypothesized that the P603A change in Np-UCR-NP2_6692 is responsible for loss of production of (-)-terremutin by NpB-UV9 mutant. This gene, conserved among fungi, is not known to be involved in (-)-terremutin biosynthesis in other fungi. Since it is not located near (-)-terremutin BGCs, it could be involved in a general regulatory network affecting the transcription of (-)-terremutin BGC genes. Such *N. parvum* infection and secondary metabolism regulatory networks have been already suggested by Massonnet et al. [36] when analyzing transcriptomic data from infected grapevines. To demonstrate our hypothesis, the level of expression of the (-)-terremutin BGC genes should be assessed in NpB-UV9 and its complemented transformant carrying the wild-type Np-B allele of Np-UCR-NP2_6692. 

### 4.4. Modulation of Host Plant Defenses by N. parvum and Its Phytotoxins (R)-Mellein and (-)-Terremutin

Leaves of cuttings infected by *N. parvum* (Figure 4) displayed an up-regulation of SA-responsive genes (*PR2*, *PR5* and *PR10*), especially upon infection with the most aggressive Np-Bt67 isolate (3.6 to 9.4-fold compared to mock). In contrast, the expression of JA/ET responsive genes was either unaffected (*LOX9*) or moderately up-regulated (*PR3*, *PR4*) upon *N. parvum* infection (2.5 to 3.0-fold), as reported by other authors [70,71,72,73]. Plant defenses relying on SA signaling are normally not efficient against necrotrophic fungi [74]. However, its induction by *N. parvum* could antagonize the expression of JA-responsive defenses [74] facilitating disease development. Since the expression of the SA-responsive gene *PR1* was only down-regulated during the infection of cuttings by the less aggressive isolate Np-B (0.4-fold) compared to mock or Np-Bt67 infection (1-fold), we hypothesize that repression of *PR1* by Np-B has a negative effect on its aggressiveness. Genes encoding enzymes involved in the biosynthesis of antimicrobial phytoalexins (*PAL*, *STS*) and in detoxification (*GST1*) were more up-regulated upon infection with Np-Bt67 and Np-B than NpB-UV9 (1.3 to 2.7-fold, Figure 4). Since only *GST1* was up-regulated by 11-fold in response to (-)-terremutin treatment (Figure 5), we hypothesize that *GST1* expression was induced by the (-)-terremutin produced by Np-Bt67 and Np-B. This up-regulation could contribute to (-)-terremutin detoxification. Now considering the phenylpropanoid pathway and derivatives, the fact that *Botryosphaeriaceae* are known to metabolize grapevine phytoalexins [19] suggests that the weak up-regulation of *PAL* and *STS* could only slightly benefit to the pathogen metabolism [15]. Phytoalexin production could also increase consecutively to a *CHI* downregulation, as observed in plantlets treated with (-)-terremutin and in infected cuttings. This suggests that this phytotoxin could inhibit the expression of the flavonoid pathway to favor the stilbenoids pathway and consequently enhance pathogen fitness. The JA/ET-responsive genes (*PR3* and *PR4*), as well as *PR2*, *PR5* and *PR10*, were slightly overexpressed in infected cuttings (Figure 4, 2.6 to 9.4-fold) but were not at all or weakly induced in plantlets treated with phytotoxins (Figure 5, 1.18 to 2.18-fold). We suggest that *GST1, PR2*, *PR3*, *PR4*, *PR5* and *PR10*, are useful defenses against *N. parvum* isolates producing (-)-terremutin. 

We hypothesized that (-)-terremutin may facilitate the Np-Bt67 disease development in grapevine cuttings by antagonizing the expression of its JA-responsive defenses. To support this hypothesis, we used the plant model *A. thaliana*. Compared to the positive control MeJA, (-)-terremutin alone did not induce the expression of *AtVSP* (Figure S1), whereas the simultaneous supply of MeJA and (-)-terremutin reduced *AtVSP* expression (Figure S1). These results support the hypothesis that (-)-terremutin contributes to weaken the expression of the host JA-dependent-defenses. Our results suggest that the production (-)-terremutin by *N. parvum* supports its development in infected grapevine tissues by interfering with host defenses. 

## Figures and Tables

**Table 1:** Genomic differences between wild-type *N. parvum* isolate Np-B and its UV mutant NpB-UV9. The genomes of wild-type *N. parvum* isolate Np-B and its UV mutant NpB-UV9 were sequenced using Illumina NGS. Sequencing reads were mapped against *N. parvum* reference genome from isolate Np-UCR-NP2 [57]. Fourteen nucleotides differences were detected between Np-B and NpB-UV9, but only two were located in coding sequences (boxes coloured purple, compared to the yellow boxes). One mutation was detected in Np-UCR-NP2_6692 encoding a putative Zn(2)-Cys6 transcription factor and the other in Np-UCR-NP2_9007 encoding a putative regulator of G protein signaling highly similar (78%) to the flbA protein of *Aspergillus nidulans* [68]. Np-UCR-NP2_6692 mutation affected the protein sequence (P603A), while Np-UCR-NP2_9007 mutation was located in an intron and is likely silent (Table 1).

Scaffold	Position	Gene Annotation	Np-UCR-NP2	Np-UCD-646-So	Np-B	NpB-UV9	AA Mutation	First Blastp Hit
UCR-NP2_v3	UCR-NP2_v3	UCR-NP2_v3						
KB916432.1	64503	UCR-NP2_6692	C	C	C	G	P603A	transcription factor cys6 protein [*Diplodia corticola*]
KB916738.1	76864	UCR-NP2_9007	C	C	C	T	intron	regulator of G protein signaling [*Botryosphaeria dothidea*]

## Data Availability

The whole genome sequences of the strains Np-B and Np-Bt67 have been deposited in the SRA NCBI database with the accession numbers SRX13878974 and SRX13878975, respectively. The sequences of genes subjected to re-annotation in this study have been deposited in the Genbank NCBI database. Sequences of genes *Np*-*atB*, *Np*-*atE*, *Np*-*atD*, UCR-NP2_6692_v2, and UCR-NP2_9007_v2 of strain Np-B have the accession numbers OM373092, OM373094, OM373096, OM677640, and OM677642, respectively. Sequences of genes *Np*-*atB*, *Np*-*atE*, *Np*-*atD*, UCR-NP2_6692_v2, and UCR-NP2_9007_v2 of strain Np-Bt67 have the accession numbers OM373093, OM373095, OM373097, OM677641, and OM677643, respectively.

## Supplementary Materials

The following supporting information can be downloaded at: https://www.mdpi.com/article/10.3390/jof8030319/s1. Figure S1: Effect of (-)-terremutin on the expression of *Arabidopsis thaliana* gene *AtVSP*, a Jasmonate pathway marker gene. Compared to the positive control MeJA, (-)-terremutin alone did not induce the expression of *AtVSP*, whereas the simultaneous supply of MeJA and (-)-terremutin reduced *AtVSP* expression. Figure S2: Phylogenic tree of fungal PKS related to Np-UCR-NP2_4356 terremutin synthase *Np-atX* and *Np*-*UCR-NP2_6207*_mellein synthase from *N. parvum*. Np-UCR-NP2_6207 protein corresponds to a *N. parvum* PKS with 95% similarities with the mellein synthase from *P. nodorum.* Np-UCR-NP2_4356 protein (Np-atX) corresponds to a *N. parvum* PKS with 65 % similarities to the 6-MSA synthase from *A. terreus* involved in terremutin biosynthesis (https://www.ncbi.nlm.nih.gov/protein/BAA20102.2?report=genbank&log$=prottop&blast_rank=25&RID=02GB07MS016, accessed on 1 July 2020). Both protein sequences were used to search for related fungal proteins using BlastP at NCBI with default settings. The 20 first non-redundant hits were recovered and aligned using MUSCLE at phylogeny.fr. The phylogenetic tree was constructed using default parameters at phylogeny.fr [64]. Np-UCR-NP2_6207 belongs to cluster of fungal PKS that is distinct from the cluster of the 6-MSA synthases involved in terremutin biosynthesis, although Np-UCR-NP2_6207 was wrongly assigned to 6-MSA synthases during *N. parvum* genome annotation. Figure S3: Phylogenic tree of *N. parvum* isolates used in this study. Available genome sequences of *N. parvum* isolates Np-UCR-NP2 and Np-UCD-646-So, and Np-Bt67 and Np-B genome sequences produced in the framework of this study were used to extract sequences from housekeeping genes actin, alpha-tubulin, beta-tubulin, calmodulin, EF1, EF2, EF3, RPB1, and RPB2. The phylogenetic tree was constructed using default parameters at phylogeny.fr [64]. Np-B/Np-UCD-646-So and Np-Bt67/Np-UCR-NP2 clustered as separate clades. Np-B and Np-UCD-646-So are closely related. Figure S4: Genomic differences detected between Np-B and Np-Bt67 *N. parvum* isolates. The genomes of wild-type *N. parvum* isolates Np-B and Np-Bt67 were sequenced using Illumina NGS. Sequencing reads were mapped against *N. parvum* reference genome from isolate Np-UCR-NP2 [57]. Variant calling from alignment files was performed with Freebayes 1.1.0-60-gc15b070 [61], with the ploidy parameter set to 1. SnpSift 4.3t [62] was used to detect variants. Most variants corresponded to SNPs. Overall, Np-B and Np-Bt67 differed by 305.339 SNPs and 76.823 indels (B). Table S1: Biosynthetic Gene clusters (BGCs) involved in secondary metabolism (SM) identified in the genome of *N. parvum* isolate Np-UCR-NP2-v3. The genome sequence of the reference isolate Np-UCR-NP2-v3 was analyzed with AntiSMASH fungal version, with defaults parameters [65]. *N. parvum* BGCs with similarity to known BGCs of 100% are highlighted in green, while those with a similarity between 30 and 90% are highlighted in yellow. A total of 60 BGCs were detected. Ten BGCs carried a single gene encoding a NRPS, ten a NRPS-like, 18 a PKS, and 11 a terpene synthase, while other BGCs carried combinations of NRPS, PKS, hybrid PKS-NRPS, and terpene synthase. A total of 11 BCGs have significant similarities (>30%, protein level) with SM gene clusters from other fungi (dimethylcoprogen, DHN melanin, patulin, alternapyrone, pyranonigrin, ilicicolin, mellein and terremutin/terreic acid). Table S2: Polymorphisms identified in genes involved in terremutin and mellein biosynthesis between *N. parvum* isolates. Contig KB916132.1 and KB916367.1, encoding respectively (A) proteins with similarities to enzymes involved in the biosynthesis of terremutin/terreic acid in *A. terreus* and (B) another PKS with 95% similarities with the mellein synthase from *P. nodorum* from reference genome UCR-NP2 that was used to map reads from genome of the isolates Np-B, Np-Bt67, Np-UCR-NP2, and Np-UCD-646-So. Polymorphisms leading to amino acid changes between proteins of isolates Np-B and Np-Bt67 are indicated by colored boxes (yellow). Table S3: Primer sequences used for qRT-PCR analysis of grapevine defense-related genes. Table S4: Amino acids sequences of proteins from genes subjected to re-annotation in this study (Np-atA, Np-atE, Np-atD, UCR-NP2_6692_v2 and UCR-NP2_9007_v2) for strains Np-B and Np-Bt67.
