# Supplementary material for: Diversity of Neofusicoccum parvum for the Production of the Phytotoxic Metabolites (-)-Terremutin and (R)-Mellein
Source: J Fungi (Basel). 2022 Mar 19;8(3):319. doi: 10.3390/jof8030319 (PMC8948911; doi:10.3390/jof8030319)
Supplement: Supplementary file 1 [file jof-08-00319-s001.zip › jof-1615084-SI.pdf]

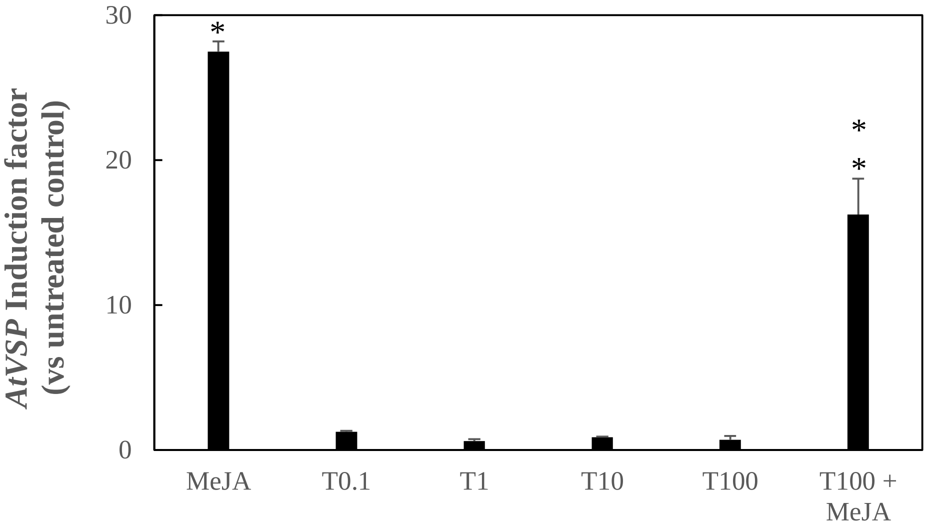

AtVSP Induction factor
(vs untreated control)
30
20
10
0
*
*
*
MeJA
T0.1
T1
T10
T100
T100 +
MeJA

Figure S1

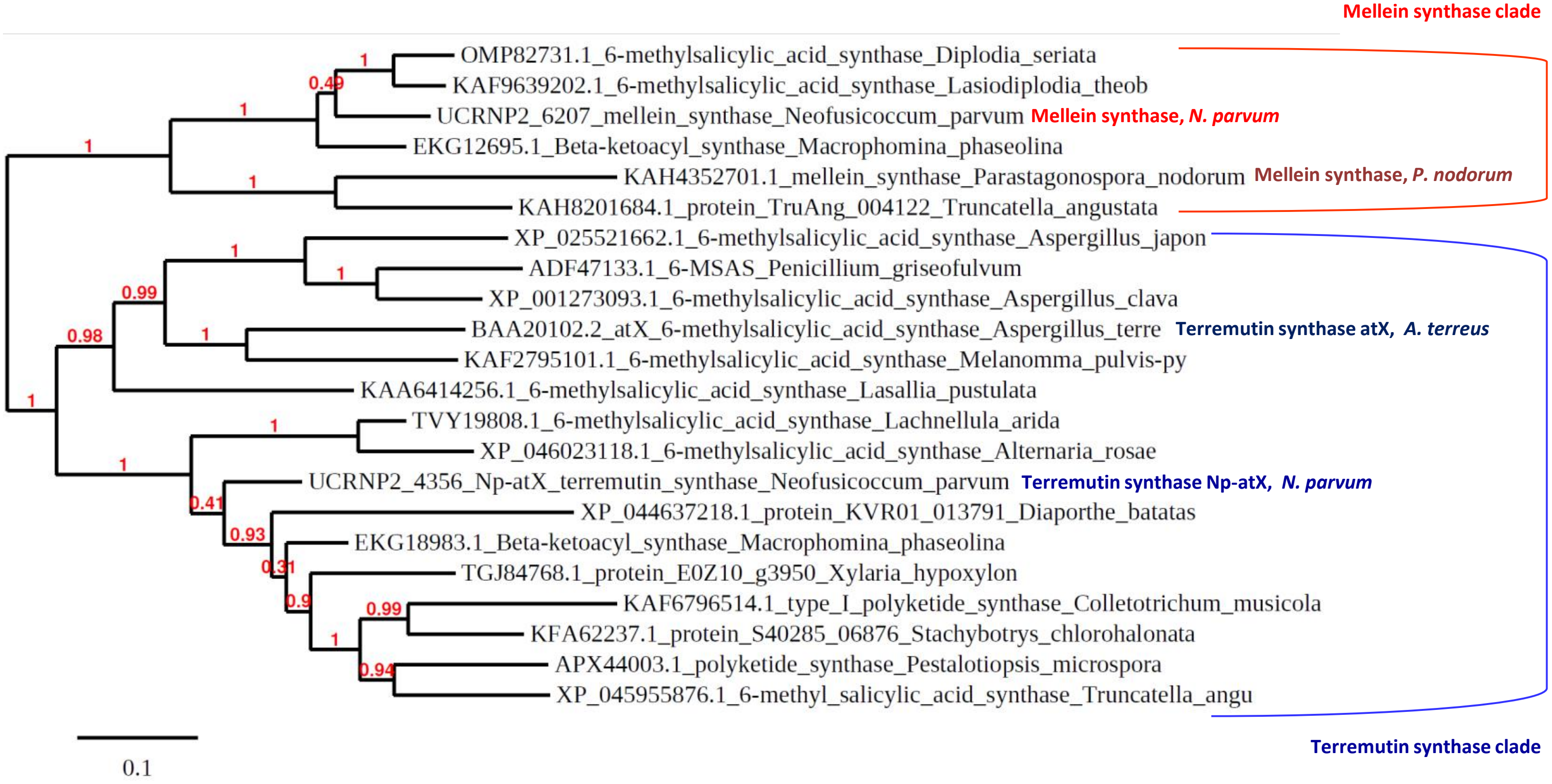

Mellein synthase clade
OMP82731.1_6-methylsalicylic_acid_synthase_Diplodia_seriata
KAF9639202.1_6-methylsalicylic_acid_synthase_Lasiodiplodia_theob
UCRNP2_6207_mellein_synthase_Neofusicoccum_parvum
Mellein synthase, N. parvum
EKG12695.1_Beta-ketoacyl_synthase_Macrophomina_phaseolina
KAH4352701.1_mellein_synthase_Parastagonospora_nodorum
Mellein synthase, P. nodorum
KAH8201684.1_protein_TruAng_004122_Truncatella_angustata
XP_025521662.1_6-methylsalicylic_acid_synthase_Aspergillus_japon
ADF47133.1_6-MSAS_Penicillium_griseofulvum
XP_001273093.1_6-methylsalicylic_acid_synthase_Aspergillus_clava
BAA20102.2_atX_6-methylsalicylic_acid_synthase_Aspergillus_terre
Terremutin synthase atX, A. terreus
KAF2795101.1_6-methylsalicylic_acid_synthase_Melanomma_pulvis-py
KAA6414256.1_6-methylsalicylic_acid_synthase_Lasallia_pustulata
TVY19808.1_6-methylsalicylic_acid_synthase_Lachnellula_arida
XP_046023118.1_6-methylsalicylic_acid_synthase_Alternaria_rosae
UCRNP2_4356_Np-atX_terremutin_synthase_Neofusicoccum_parvum
Terremutin synthase Np-atX, N. parvum
XP_044637218.1_protein_KVR01_013791_Diaporthe_batatas
EKG18983.1_Beta-ketoacyl_synthase_Macrophomina_phaseolina
TGJ84768.1_protein_E0Z10_g3950_Xylaria_hypoxylon
KAF6796514.1_type_I_polyketide_synthase_Colletotrichum_musicola
KFA62237.1_protein_S40285_06876_Stachybotrys_chlorohalonata
APX44003.1_polyketide_synthase_Pestalotiopsis_microspora
XP_045955876.1_6-methyl_salicylic_acid_synthase_Truncatella_angu
Terremutin synthase clade
0.1

Figure S2

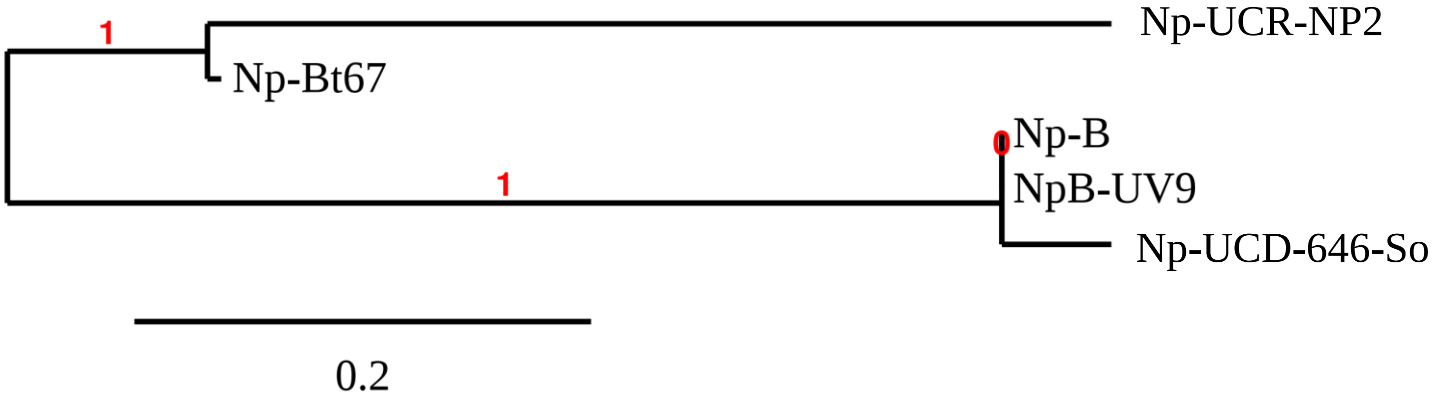

Figure S3

A

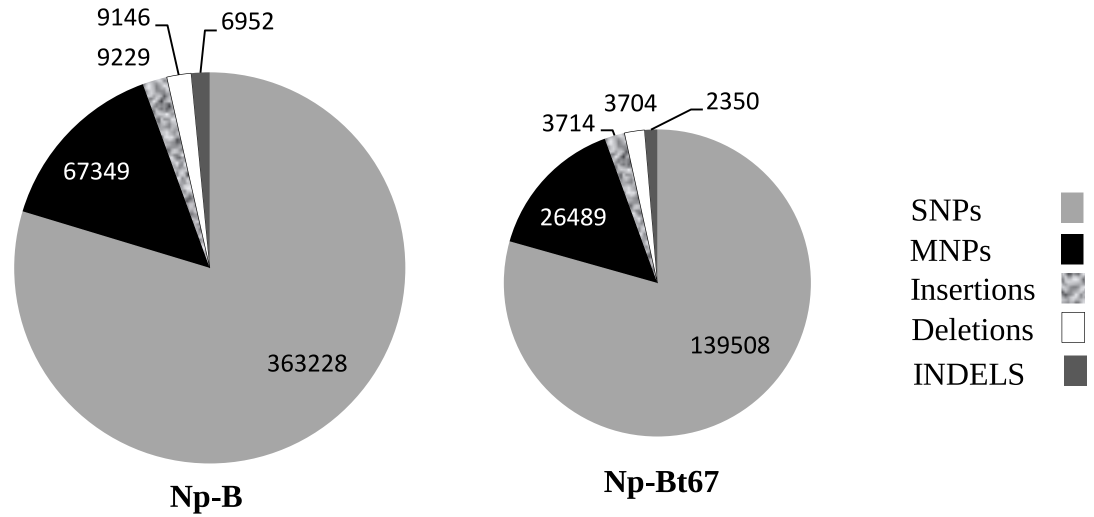

B

Genomic differences between Np-B and Np-Bt67	
SNPs differences after filtering (*)	305339
INDELs and MNPs differences after filtering (*)	76823

(*) major allele read count frequency ≥ 0.8

Figure S4

Table S1. Biosynthetic Gene clusters (BGCs) involved in secondary metabolism (SM) identified in the genome of *N. parvum* isolate Np-UCR-NP2-v3. The genome sequence of the reference isolate Np-UCR-NP2-v3 was analyzed with AntiSMASH fungal version, with defaults parameters [65]. N. parvum BGCs with similarity to known BGCs of 100% are highlighted in green, while those with a similarity between 30 and 90% are highlighted in yellow. 60 BGCs were detected. Ten BGCs carried a single gene encoding a NRPS, ten a NRPS-like, 18 a PKS, and 11 a terpene synthase, while other BGCs carried combinations of NRPS, PKS, hybrid PKS-NRPS, and terpene synthase. 11 BCGs have significant similarities (>30%, protein level) with SM gene clusters from other fungi (dimethylcoprogen, DHN melanin, patulin, alternapyrone, pyranonigrin, ilicicolin, mellein and terremutin/terreic acid).

Contig	Region	Type	From	To	Most similar known cluster	Key gene type	Similarity
KB916495.1	Region 722.1	betalactone	49,501	72,006			
KB916648.1	Region 847.1	betalactone	44,357	75,083			
KB915688.1	Region 42.1	NRPS	1	27,915			
KB915768.1	Region 112.1	NRPS	47,393	105,202			
KB915926.1	Region 255.1	NRPS	1	53,154	dimethylcoprogen	NRP	100%
KB915996.1	Region 307.1	NRPS	1	46,294			
KB916027.1	Region 335.1	NRPS	5,013	43,666			
KB916118.1	Region 416.1	NRPS	1	40,624			
KB916185.1	Region 472.1	NRPS	34,514	91,064			
KB916208.1	Region 487.1	NRPS	11,386	83,786	leucinostatin A / leucinostatin B	Polyketide	10%
KB916760.1	Region 944.1	NRPS	10,429	36,515	hexadehydroastechrome / terezine-D / astechrome	NRP	37%
KB916846.1	Region 1012.1	NRPS	31,26	81,782			
KB916008.1	Region 317.1	NRPS,T1PKS	1	44,985			
KB916424.1	Region 662.1	NRPS,T1PKS	1	34,504	equisetin	NRP + Polyketide	27%
KB916836.1	Region 1006.1	NRPS,T1PKS	1,452	46,922	pyranonigrin E	Polyketide	100%
KB916836.1	Region 1006.2	NRPS,T1PKS	61,37	111,856	ilicicolin H	NRP + Polyketide	50%
KB916847.1	Region 1013.1	NRPS,T1PKS	4,136	55,628	oxaleimide C	NRP + Polyketide	10%
KB915669.1	Region 25.1	NRPS-like	1	14,044			
KB915971.1	Region 288.1	NRPS-like	70,345	109,121			
KB915983.1	Region 297.1	NRPS-like	27,278	60,046			
KB916221.1	Region 499.1	NRPS-like	1	13,308			
KB916267.1	Region 534.1	NRPS-like	21,37	44,106			
KB916317.1	Region 570.1	NRPS-like	135,414	177,953			
KB916317.1	Region 570.2	NRPS-like	200,878	238,304			
KB916340.1	Region 590.1	NRPS-like	49,756	93,519			
KB916548.1	Region 764.1	NRPS-like	76,825	120,776			
KB916667.1	Region 864.1	NRPS-like	1,293	44,944			
KB915946.1	Region 270.1	NRPS-like,T1PKS	6,252	69,735			
KB915668.1	Region 24.1	T1PKS	12,15	68,081	azanigerone A	Polyketide	33%
KB915702.1	Region 54.1	T1PKS	1	34,025			
KB915787.1	Region 127.1	T1PKS	10,302	44,913			
KB915792.1	Region 131.1	T1PKS	9,413	57,45			
KB915896.1	Region 226.1	T1PKS	1	43,074			
KB915905.1	Region 235.1	T1PKS	1	15,47	4-epi-15-epi-brefeldin A	Polyketide:Iterative type I	20%
KB915930.1	Region 259.2	T1PKS	229,692	276,533			
KB916132.1	Region 428.1	T1PKS	10,011	55,459	terremutin/terreic acid	Polyketide	44%
KB916202.1	Region 483.1	T1PKS	225,506	273,291			
KB916240.1	Region 512.1	T1PKS	58,487	105,101	melanin	Polyketide	100%
KB916367.1	Region 616.1	T1PKS	1	32,761	(-)-mellein	Polyketide	100%
KB916530.1	Region 750.1	T1PKS	1	27,781	alternapyrone	Polyketide	60%
KB916578.1	Region 792.1	T1PKS	13,962	45,733			
KB916620.1	Region 826.1	T1PKS	1	39,978			
KB916717.1	Region 905.1	T1PKS	1	36,465			
KB916726.1	Region 912.1	T1PKS	1	24,503	patulin	Polyketide:Iterative type I	33%
KB916773.1	Region 957.1	T1PKS	61,679	108,565			
KB916895.1	Region 1049.1	T1PKS	1	15,355			
KB916776.1	Region 959.1	T1PKS,NRPS	357	39,898			
KB916727.1	Region 913.1	T1PKS,NRPS-like	1	29,301			
KB915641.1	Region 3.1	terpene	1	12,059	heptelidic acid	Terpene	27%
KB915845.1	Region 179.1	terpene	43,203	68,594			
KB915870.1	Region 202.1	terpene	32,886	57,525			
KB915879.1	Region 210.1	terpene	77,919	103,069			
KB915930.1	Region 259.1	terpene	155,925	177,677			
KB915943.1	Region 267.1	terpene	6,631	27,737			
KB916055.1	Region 361.1	terpene	16,695	39,939	dimethylcoprogen	Terpene	100%
KB916504.1	Region 729.1	terpene	129,991	153,157			
KB916581.1	Region 794.1	terpene	16,376	37,893			
KB916741.1	Region 926.1	terpene	15,235	46,972			
KB916766.1	Region 950.1	terpene	182,981	204,139			
KB916334.1	Region 585.1	terpene,T1PKS	29,649	79,29			

Table S2. Polymorphims identified in genes involved in terremutin and mellein biosynthesis between *N. parvum* isolates. Contig KB916132.1 and KB916367.1, encoding respectively (A) proteins with similarities to enzymes involved in the biosynthesis of terremutin/terreic acid in *A. terreus* and (B) another PKS with 95% similarities with the mellein synthase from *P. nodorum* from reference genome UCR-NP2 that was used to map reads from genome of the isolates Np-B, Np-Bt67, Np-UCR-NP2 and Np-UCD-646-So. Polymorphisms leading to amino-acid changes between proteins of isolates Np-B and Np-Bt67 are indicated by colored boxes (yellow).

Scaffold	Gene	Nucleotide variant position in scaffold	AA variant Position in peptide	Np-B	Np-Bt67	Np-UCR-NP2	Np-UCD-646-So
(A)	Np-atF (UCR-NP2_4355) transcription factor	28191	17	S	S	N	S
		27821	122	K	K	R	K
		27579	203	P	P	A	P
		27479	236	N	N	T	N
		26303	628	I	I	T	I
		26020	723	G	-	-	G
		25960	743	E	D	D	E
KB916132.1 terremutin cluster	Np-atX (UCR-NP2_4356) PKS 6-MSA synthase	33987	472	D	G	G	D
		32532	957	S	N	N	S
		31693	1237	A	T	T	A
		31660	1248	A	T	T	A
		31624	1260	N	N	N	T
		31081	1441	VASFLVEKGARRLVLLSR RGLPPRREWK	VASFLVEKGARRLVLLSR RGLPPRREWK	VASFLVEKGARRLVLLSR RGLPPRREWK	-
		30781	1541	A	A	P	A
		30301	1701	T	T	A	T
	Np-atB (UCR-NP2_4357) MFS	38579	11	E	D	D	E
	Np-atE (UCR-NP2_4359.1) P450	45122	9	V	V	A	V
		45056	31	T	I	T	T
	Np-atD (UCR-NP2_4359.2) epoxidase	46760	55	D	E	E	D
(B)	UCR-NP2_6207, PKS Mellein_Synthase	8248	420	K	Q	Q	K
		8368	460	R	G	G	R
		8380	464	A	P	P	A
		8384	465	A	E	E	A
KB916367.1 Mellein synthase		9578	863	T	K	K	T
		10168	1060	V	L	V	V
		10520	1177	N	S	N	N
		10533	1181	E	D	D	E
		10702	1238	V	L	L	V
		10960	1324	I	V	V	I
		11079	1363	Q	H	H	Q

Table S3: Primer sequences used for qRT-PCR analysis of grapevine defense-related genes.

Gene	Name	Accession number[1]	Forward Primer (5'-3')	Reverse primer (5'-3')	Annealing temperature (°C)	Amplicon size (bp)	Efficiency of primers pairs (%)
60RSP	60S ribosomal protein L18	XM_002270599[1]	ATCTACCTCAAGCTCCTAGTC	CAATCTTGTCCTCCTTTCCT	60	166	100.0
EF1	elongation factor 1-alpha	XM_002284888[1]	AACCAAAATATCCGGAGTAAAAGA	GAACTGGGTGCTTGATAGGC	60	164	100.0
PR3	class IV chitinase (CHI4C)	NM_001281244[1]	TCGAATGCGATGGTGGAAA	TCCCCTGTCGAAACACCAAG	60	91	99.9
PR4	PR-4 type protein	AF061329[1]	TGGTTATAGCCAGCCCCATTAG	AAGCTGCCTGTGGCAAGTG	60	151	99.2
LOX9	Lipoxygenase	NM_001281249[1]	CCCTTCTTGGCATCTCCCTTA	TGTTGTGTCCAGGGTCCATTC	60	101	90.0
PR1	pathogenesis-related protein 1	XM_002273752[1]	GGAGTCCATTAGCACTCCTTTG	CATAATTCTGGGCGTAGGCAG	60	168	90.0
PR2	Class I beta-1,3-glucanase	NM_001280967[1]	TCAATGGCTGCAATGGTGC	CGGTCGATGTTGCGAGATTTA	60	155	97.2
PR5	thaumatin-like protein	XM_002282994[1]	TTTGATCATCCTTAGGGTAGCTGTAA	ACCAATGAGTACTGTTGCAATTCC	60	106	97.6
GST1	glutathione S-transferase	NM_001281248[1]	TGCATGGAGGAGGAGTTCGT	CAAGGCTATATCCCCATTTTCTTC	60	98	90.0
PR10	pathogenesis-related protein 10.3	DQ396809[1]	CGTTAAGGGCGGCAAAGAG	GCATCAGGGTGTGCCAAGA	60	75	93.5
PAL	phenylalanine ammonia lyase	XM_003635637[1]	TCCTCCCGGAAAACAGCTG	TCCTCCAAATGCCTCAAATCA	60	101	92.9
STS	stilbene synthase	NM_001281117[1]	AGGAAGCAGCATTGAAGGCTC	TGCACCAGGCATTTCTACACC	60	101	94.3
CHI	Chalcone isomerase	NM_001281104[1]	GCAGAAGCCAAAGCCATTGA	GCCGATGATGGACTCCAGTAC	60	201	101.5

[1] NCBI accession number

Table S4: Amino acids sequences of proteins from genes subjected to re-annotation in this study (Np-atA, Np-atE, Np-atD, UCR-NP2_6692_v2, UCR-NP2_9007_v2) **for strains Np-B and Np-Bt67.**

Np-atB gene, Np-B strain
Genbank accession ID : OM373092

>**Np-atB_Np-B**
MSIKSGSSTGEASNSKTSTPNHATPIRGSADEKQQDPEANGEPQWDPAFEVSFSGDTDPRNPKSMSELRK
WFIVIIVATTSLCVACTSSLYTGTYAQVEAEFGSRRVITTLGLSMFVVGLGLSPMVLAPLCEFYGRKPVYIASTF
FFVVWIIPCAVAKNITTLVIARFLDGFAGAAFLSVAGGTVGDLFTKSTLQAPMMVYTASPFLGPELGPVIGNFIN
YYVNWRWTFWVLLIWAGVQFLLVLFCVPETYSPVLLRKEAQKMRKETGDDRWHAPIEKMKRSVGGTILRSC
YRPFMLLLLEPMVLLLCTFCAVVLGVLYLFFGAFNLVFTNNHNFNHWQVGLSFLGIMVGMVIGILSDPLWKKN
YLRLLENHKKRTGEAGSEPEYRLAPSIVGAPLVVIGLFWFGWTQYSFIHWMVPIVGSAFFGCGIILVFSGIFTFL
VESYPLYAASALAANSFARSMFAAMFPLFGPAMYNNLGYQWATFLLTMICCVLAPFPYIFYKYGKRIRKRSRY
VGAR

Np-atB gene, Np-Bt67 strain
Genbank accession ID : OM373093

>**Np-atB_Np-Bt67**
MSIKSGSSTGDASNSKTSTPNHATPIRGSADEKQQDPEANGEPQWDPAFEVSFSGDTDPRNPKSMSELRK
WFIVIIVATTSLCVACTSSLYTGTYAQVEAEFGSRRVITTLGLSMFVVGLGLSPMVLAPLCEFYGRKPVYIASTF
FFVVWIIPCAVAKNITTLVIARFLDGFAGAAFLSVAGGTVGDLFTKSTLQAPMMVYTASPFLGPELGPVIGNFIN
YYVNWRWTFWVLLIWAGVQFLLVLFCVPETYSPVLLRKEAQKMRKETGDDRWHAPIEKMKRSVGGTILRSC
YRPFMLLLLEPMVLLLCTFCAVVLGVLYLFFGAFNLVFTNNHNFNHWQVGLSFLGIMVGMVIGILSDPLWKKN
YLRLLENHKKRTGEAGSEPEYRLAPSIVGAPLVVIGLFWFGWTQYSFIHWMVPIVGSAFFGCGIILVFSGIFTFL
VESYPLYAASALAANSFARSMFAAMFPLFGPAMYNNLGYQWATFLLTMICCVLAPFPYIFYKYGKRIRKRSRY
VGAR

Np-atE gene, Np-B strain
Genbank accession ID : OM373094

>**Np-atE_Np-B**
MESPSSLVVPPISLHREHFFAVSLFLFVLATIVSALRIGKREKNLPPGPPTIPLLGNEHLIPKADGHFIMGKWAK
EYGGIFSLKRFMNTTLVINDWKYVKSLLDKKSTLYSYRPKSLVADLITRGDHILMMQYGDTWRTVRKLIHQTF
MESNCDKYHYKVQEAEANQMIYDFMVNPADHMSHPKRFSNSITMSLVFGFRTKSVNDDYLRRLYHAMEKW
SLVLETGATPPVDSFPLLKLIPERFMGNWRSRAIECGDLMRDLYGEVLEKVRQRRQQGINKGSLMDKVLDQQ
SKYNFNDHQLAFFGGTLMEGGSDTSSSLVLAIIQAMTQYPEVQKRAQAEIDSVVGFDRSPRWSDFSKLPYIN
MIIKEAHRWRPVLPLGVVHAVAQDDTVDGMHLPKESTVIINVWALHNDEKRWKSPEDFIPERYETYPELAPFY
AASKDFDKRDHLGYGASRRICPGIHLAERNLFIAAAKLLWAFDFAVDPNMKNDASAETGSSQGFMHCVKDYE
CKITLRDERKRETILRELADAQPIFAQYE

Np-atE gene, Np-Bt67 strain
Genbank accession ID : OM373095

>**Np-atE_Np-Bt67**
MESPSSLVVPPISLHREHFFAVSLFLFVLAIIVSALRIGKREKNLPPGPPTIPLLGNEHLIPKADGHFIMGKWAKE
YGGIFSLKRFMNTTLVINDWKYVKSLLDKKSTLYSYRPKSLVADLITRGDHILMMQYGDTWRTVRKLIHQTFM
ESNCDKYHYKVQEAEANQMIYDFMVNPADHMSHPKRFSNSITMSLVFGFRTKSVNDDYLRRLYHAMEKWSL
VLETGATPPVDSFPLLKLIPERFMGNWRSRAIECGDLMRDLYGEVLEKVRQRRQQGINKGSLMDKVLDQQS
KYNFNDHQLAFFGGTLMEGGSDTSSSLVLAIIQAMTQYPEVQKRAQAEIDSVVGFDRSPRWSDFSKLPYINM
IIKEAHRWRPVLPLGVVHAVAQDDTVDGMHLPKESTVIINVWALHNDEKRWKSPEDFIPERYETYPELAPFYA
ASKDFDKRDHLGYGASRRICPGIHLAERNLFIAAAKLLWAFDFAVDPNMKNDASAETGSSQGFMHCVKDYEC
KITLRDERKRETILRELADAQPIFAQYE

Np-atD gene, Np-B strain
Genbank accession ID : OM373096

>**Np-atD_Np-B**
MPLFTPFVPYHVSAGQNEIKKFGGLLTTEWLPPLPGRSFRLRQTYRKTIDGPIPDNLRKLINSPGRPKGPPTH
FHQWQTEYFTVEHGVCAVEIDGKVTKLTPADGEVSVKPGHIHAFWLDDETPDYMTVILSATDSGKDYQLDRV
FFENWYGYWHDALLYEGGLDLIQTLCTHDAGDHYTPAPKWVPFRRFFGYWGCVIIGRWLGGMLGYKPFFKE
YTTDWDYAVEKMNGSFWQRRLADKSYTHRTTWDRQVELSQGPDPRNAEYQDMVTDLAETRDPSSKIETAG
LPIPVAGPEGPFWGKVALNGTRVDSVSGISSGFEVNGTGKAAALSQRREHS

Np-atD gene, Np-Bt67 strain
Genbank accession ID : OM373097

>**Np-atD_Np-Bt67**
MPLFTPFVPYHVSAGQNEIKKFGGLLTTEWLPPLPGRSFRLRQTYRKTIDGPIPENLRKLINSPGRPKGPPTH
FHQWQTEYFTVEHGVCAVEIDGKVTKLTPADGEVSVKPGHIHAFWLDDETPDYMTVILSATDSGKDYQLDRV
FFENWYGYWHDALLYEGGLDLIQTLCTHDAGDHYTPAPKWVPFRRFFGYWGCVIIGRWLGGMLGYKPFFKE
YTTDWDYAVEKMNGSFWQRRLADKSYTHRTTWDRQVELSQGPDPRNAEYQDMVTDLAETRDPSSKIETAG
LPIPVAGPEGPFWGKVALNGTRVDSVSGISSGFEVNGTGKAAALSQRREHS

UCR-NP2_6692_v2, Np-B strain
Genbank accession ID : OM677640

>**UCR-NP2_6692_v2_Np-B**
MPAKRPPPSGATPGSTTKHPKTEHQSSDDFSSSVKKKLSASTRTGQACDRCKVRKIRCDGRPGGCSPCVQ
NNTECKTTDRITGRATTRGHTEQLEYENNHLKGALYALQQQLKEIGVEPRIPSNPQAPQGFAPSLSPQPQSA
WQNGSHDGQLWGSTPPAPSTASLSNYPHMNDANGTRDTDQSQFHGPNLPTFRAGLHGDNYLGVSSANSV
LSPIKGTSLSFFGMEIDLNDFVPDDVEDSSNPVSYQHFLSVALNAPGCPRPKKEELPASYSECATYATWYFRG
LHPYAPVLYKPHFMELLSRIYSDPTYQPTPAEEVQVQMVLAFIKYQFSARNGNAESLKQAHAHYRYALSFFYT
LVNSHTIADVQALTMICLHLRNFPKPGAAWMMSSMTFMISVELGLHRSARAWTDMGPKRDPIEVETRKRVF
WTLHGLYIALCGRLGRPMPLRMEDIDVEFPEPIHDYLPSETNLSDFRKCSFHVGLQAIRILAVHSEMYSTIHAV
RQSPDLYATHLRRLEDEHRKWKEQVPLELREGSRADSEDYIFSLYVQFWDHEFNLMLHHPAMCRSRDPELL
NSNMSICIAATSKMVRVLDEVRKYKSLDAPWVNCTAYLAAMLTTLFVYSHRKDELTSSDLAKLKSEMDMWLLI
MDECGSILGSGKRLEKACRSIIDHTVQNISHHLAKKTASAASAVANVLKTEPDTSPRQEPTPAPSYANNNAYAS
AYGHTVNGHANVHKPSTGGYLPPDDPSLSQSSNPYPTVQAQYSSYPEQAQTRLPYQNGAPRYEQTQYNVP
GGDMNAAHVAAAASAAATQDSSAAFAYPPGSHGNFGQVTNQSDAWRQWMMTNLGPQEYHHASALMALG
HTGGHDTAVSTGADDNPGVLADMHSVAVNGLHSPHPAVSAAAVAQAAHSQQLWPLMVFDIGQQGGPNVTQ

UCR-NP2_6692_v2, Np-Bt67 strain
Genbank accession ID : OM677641

>**UCR-NP2_6692_v2_Np-Bt67**
MPAKRPPPSGATPGSTTKHPKTEHQSSDDFSSSVKKKLSASTRTGQACDRCKVRKIRCDGRPGGCSPCVQ
NNTECKTTDRITGRATTRGHTEQLEYENNHLKGALYALQQQLKEIGVEPRIPSNPQAPQGFAPSLSPQPQSA
WQNGSHDGQLWGSTPPAPSTASLSNYPHMNDANGTRDTDQSQFHGPNLPTFRAGLHGDNYLGVSSANSV
LSPIKGTSLSFFGMEIDLNDFVPDDVEDSSNPVSYQHFLSVALNAPGCPRPKKEELPASYSECATYATWYFRG
LHPYAPVLYKPHFMELLSRIYSDPTYQPTPAEEVQVQMVLAFIKYQFSARNGNAESLKQAHAHYRYALSFFYT
LVNSHTIADVQALTMICLHLRNFPKPGAAWMMSSMTFMISVELGLHRSARAWTDMGPKRDPIEVETRKRVF
WTLHGLYIALCGRLGRPMPLRMEDIDVEFPEPIHDYLPSETNLSDFRKCSFHVGLQAIRILAVHSEMYSTIHAV
RQSPDLYATHLRRLEDEHRKWKEQVPLELREGSRADSEDYIFSLYVQFWDHEFNLMLHHPAMCRSRDPELL
NSNMSICIAATSKMVRVLDEVRKYKSLDAPWVNCTAYLAAMLTTLFVYSHRKDELTSSDLAKLKSEMDMWLLI
MDECGSILGSGKRLEKACRSIIDHTVQNISHHLAKKTASAASAVANVLKTEPDTSPRQEPTPAPSYANNNAYAS
AYGHTVNGHANVHKPSTGGYLPPDDPSLSQSSNPYPTVQAQYSSYPEQAQTRLPYQNGAPRYEQTQYNVP
GGDMNAAHVAAAASAAATQDSSAAFAYPPGSHGNFGQVTNQSDAWRQWMMTNLGPQEYHHASALMALG
HTGGHDTAVSTGADDNPGVLADMHSVAVNGLHSPHPAVSAAAVAQAAHSQQLWPLMVFDIGQQGGPNVTQ

UCR-NP2_9007_v2, Np-B strain
Genbank accession ID : OM677642

>**UCR-NP2_9007_v2_Np-B**
MHQTSSRLLRMTDDDRPFTRDFKDLFSTLMVSLPLTPHRVRWQKVDHSFTSEEAMTNLGSLKFSQSNRMP
DPKDPSRIVTTTTTTTFSMAKEMARSVCQKFLEARFIESVEGKTDFMSKTAVWQMTPKGMHVLQRFCQRNGI
NQKHVYEVLDSSRNTMHLVILERELETDKLNHDQATIEVVFRRFVGHEGPNAKANTAQADSDSLNEYSTGMV
GVKMARERKIGDKIYMNTFTGKASVDWLMDCSTMVDKREAYEMCSLFYEFGLMAPVEVFPNSNRFQPTKS
AIYYVTEKGQRIAGWIQSPKASLNGDAAAAAANRPREGTTRDSNANRMTVIIRDPALRLLFREFLRETHCEEN
LSFYLDVSEFLGGYKQAKRANQTPKLEIIRETLAAAYSLYNAFLAPGSPCELNIDHSLRTALAARMTRAVGDDE
AMVRSLDEVATLFDQAQNSVFKLMASDSVPKFMREPKYASVIRDRNLDAMPGSFNNNNSAGLAAS

UCR-NP2_9007_v2, Np-Bt67 strain
Genbank accession ID : OM677643

>**UCR-NP2_9007_v2_Np-Bt67**
MHQTSSRLLRMTDDDRPFTRDFKDLFSTLMVSLPLTPHRVRWQKVDHSFTSEEAMTNLGSLKFSQSNRMP
DPKDPSRIVTTTTTTTFSMAKEMARSVCQKFLEARFIESVEGKTDFMSKTAVWQMTPKGMHVLQRFCQRNGI
NQKHVYEVLDSSRNTMHLVILERELETDKLNHDQATIEVVFRRFVGHEGPNAKANTAQADSDSLNEYSTGMV
GVKMARERKIGDKIYMNTFTGKASVDWLMDCSTMVDKREAYEMCSLFYEFGLMAPVEVFPNSNRFQPTKS
AIYYVTEKGQRIAGWIQSPKASLNGDAAAAAANRPREGTTRDSNANRMTVIIRDPALRLLFREFLRETHCEEN
LSFYLDVSEFLGGYKQAKRANQTPKLEIIRETLAAAYSLYNAFLAPGSPCELNIDHSLRTALAARMTRAVGDDE
AMVRSLDEVATLFDQAQNSVFKLMASDSVPKFMREPKYASVIRDRNLDAMPGSFNNNNNSAGLAAS
